# A role for foregut tyrosine metabolism in glucose tolerance

**DOI:** 10.1016/j.molmet.2019.02.008

**Published:** 2019-02-27

**Authors:** Judith Korner, Gary W. Cline, Mark Slifstein, Pasquale Barba, Gina R. Rayat, Gerardo Febres, Rudolph L. Leibel, Antonella Maffei, Paul E. Harris

**Affiliations:** 1 Department of Medicine and the Naomi Berrie Diabetes Center, Columbia University, College of Physicians and Surgeons, New York, NY, 10032, USA; 2 Yale Diabetes Research Center, Yale School of Medicine, New Haven, CT, 06520, USA; 3 Department of Psychiatry, Stony Brook University, Stony Brook, New York, NY, 11794, USA; 4 Institute of Genetics and Biophysics, Adriano Buzzati-Traverso, CNR, Naples, IT 80131, Italy; 5 Alberta Diabetes Institute, Ray Rajotte Surgical-Medical Research Institute, Department of Surgery, University of Alberta, Edmonton, AB, T6G 2E1 CA, Canada

**Keywords:** Dopamine, Glucose homeostasis, GSIS, Tyrosine, Gut

## Abstract

**Objective:**

We hypothesized that DA and L-DOPA derived from nutritional tyrosine and the resultant observed postprandial plasma excursions of L-DOPA and DA might affect glucose tolerance via their ability to be taken-up by beta cells and inhibit glucose-stimulated β-cell insulin secretion.

**Methods:**

To investigate a possible circuit between meal-stimulated 3,4-dihydroxy-L-phenylalanine (L-DOPA) and dopamine (DA) production in the GI tract and pancreatic β-cells, we: 1) mapped GI mucosal expression of tyrosine hydroxylase (TH) and aromatic amino acid decarboxylase (AADC); 2) measured L-DOPA and DA content of GI mucosal tissues following meal challenges with different L-tyrosine (TYR) content, 3) determined whether meal TYR content impacts plasma insulin and glucose excursions; and 4) characterized postprandial plasma excursions of L-DOPA and DA in response to meal tyrosine content in rodents and a population of bariatric surgery patients. Next, we characterized: 1) the metabolic transformation of TYR and L-DOPA into DA in vitro using purified islet tissue; 2) the metabolic transformation of orally administrated stable isotope labeled TYR into pancreatic DA, and 3) using a nuclear medicine technique, we studied endocrine beta cells in situ release and binding of DA in response to a glucose challenge.

**Results:**

We demonstrate in rodents that intestinal content and circulatory concentrations L-DOPA and DA, plasma glucose and insulin are responsive to the tyrosine (TYR) content of a test meal. Intestinal expression of two enzymes, Tyrosine hydroxylase (TH) and Aromatic Amino acid Decarboxylase (AADC), essential to the transformation of TYR to DA was mapped and the metabolism of metabolism of TYR to DA was traced in human islets and a rodent beta cell line in vitro and from gut to the pancreas in vivo. Lastly, we show that β cells secrete and bind DA in situ in response to glucose stimulation.

**Conclusions:**

We provide proof-of-principle evidence for the existence of a novel postprandial circuit of glucose homeostasis dependent on nutritional tyrosine. DA and L-DOPA derived from nutritional tyrosine may serve to defend against hypoglycemia via inhibition of glucose-stimulated β-cell insulin secretion as proposed by the anti-incretin hypothesis.

## Highlights

• Nutritional tyrosine is metabolized to L DOPA and DA in the foregut.
• Postprandial L-DOPA and DA plasma concentrations rise in response to tyrosine.
• Oral stable isotope labeled tyrosine is found postprandially in the pancreas as DA.
• L-DOPA and DA are inhibitors of beta cell glucose-stimulated insulin secretion.
• Postprandial L-DOPA and DA excursions are muted in certain bariatric surgery patients.

## Graphical abstract

**Figure:**
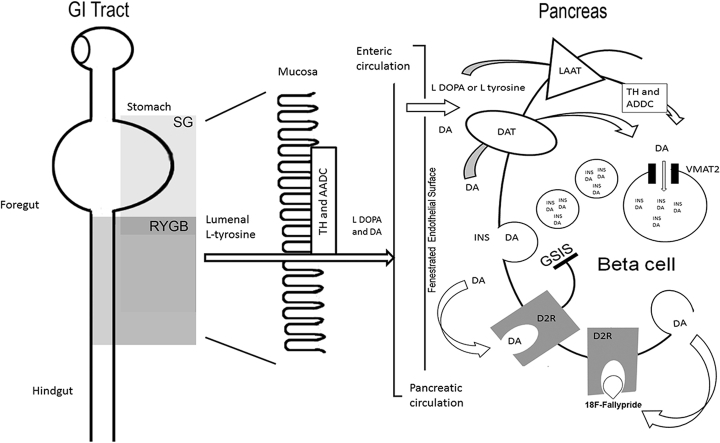

**Schematic of the proposed gut to pancreas blood glucose concentration regulatory circuit**. Abbreviations used: TH, tyrosine hydroxylase; AADC, Aromatic l-amino acid decarboxylase; L-DOPA, L-3,4-dihydroxyphenylalanine; D2R, Dopamine receptor type 2, DA, Dopamine; LAAT, L-Type Amino Acid Transporter 1; DAT, dopamine active transporter; VMAT2, Vesicular monoamine transporter type 2; GSIS, glucose-stimulated insulin secretion. Grey boxes show tissue bypassed by GS, Sleeve Gastrectomy, and RYGB, Roux-en-Y Gastric Bypass surgeries.

## 1 Introduction

Pancreatic β-cells integrate several different external signals to fine-tune the release of insulin and thus accurately regulate blood glucose levels commensurate with metabolic demand. While D-Glucose is the major physiological stimulus for insulin secretion, fatty acids and some, but not all [1], amino acids promote insulin secretion by β-cells either alone or in synergy with D-glucose. Net insulin production and glucose homeostasis are regulated by a number of other molecules as well, including several gut-derived peptide hormones (e.g. GLP-1) and a number of classical neurotransmitters (e.g. epinephrine). Many of these molecules function as amplifying agents (“incretins”) that, although they have little or no effect alone, enhance the signals generated by the β-cell glucose sensing apparatus (reviewed in [2]).

Mesenteric organs synthesize the majority of the total “classical” neurotransmitter dopamine and L-DOPA in the body from its biosynthetic precursor Tyrosine (TYR) [3]. The foregut (including the stomach) is the major source of secreted and circulating DA and L-DOPA [4], [5]. After ingestion of a standard mixed meal (MMTT), healthy human volunteers, as well as rodents, show up to two-fold over baseline increases in plasma DA and L-DOPA concentrations [6], [7]. The kinetics of DA and L-DOPA excursions coincide with postprandial GLP-1 levels observed after a mixed meal tolerance test (MMTT) administered to Lewis rats [8].

Both L-DOPA and DA inhibit glucose-stimulated insulin secretion (GSIS) by human and rodent islet β-cells in vitro [9], [10], [11], [12], [13]. β-cells take-up L-DOPA and transform it to DA and are also the major storage depot of pancreatic DA [11], [14]. β-cells can import and store DA in insulin storage vesicles [8], [12] via the action of DA active transporter (DAT). β-cells co-secrete DA as well as insulin in response to glucose stimulus and express Vesicular Monoamine transporter type 2 (VMAT2), dopamine 2 like receptors (D2R), and the large aromatic amino acid transporter, LAT1, responsible for the import of L-DOPA [8], [12], [15]. Experiments with dopamine antagonists suggest that the co-secreted dopamine binds to D2R to down-regulate β-cell insulin secretion [12]. Most recently, murine β-cell-specific knockout of D2R or D3R lose the phenotype of DA-mediated inhibition of GSIS in vivo [13].

The exact mechanism(s) underlying the reversal of hyperglycemia in response to bariatric surgery is currently unknown, but several biochemical mechanisms accounting for improvements of glucose homeostasis with Roux-en-Y gastric bypass surgery (RYGB) independent of weight loss have been proposed (reviewed in [16], [17]). Two are of particular interest; a) the “hindgut hypothesis” suggesting that nutrient delivery to the distal intestine drives the production of “incretins” which enhance insulin secretion (e.g. GLP-1), and b) the “foregut hypothesis.” The foregut hypothesis proposes that foregut bypass reduces the elaboration of nutrient-stimulated intestinal signals (e.g. anti-incretins) that limit insulin secretion, and normally defend against hypoglycemia thereby maintaining homeostasis [18], [19], [20]. We set out to examine the role of nutritional TYR as a source of possible “anti-incretins” in healthy rodents.

## 2 Materials and methods

### 2.1 Materials

Table S1 contains information regarding the source of reagents and equipment used in these studies.

### 2.2 Human studies

The Columbia University Institutional Review Board approved the study; written informed consent was obtained from all patients. Participants were recruited from patients who presented for surgical treatment of obesity. Standard criteria for performing bariatric surgery were used for selection of obese patients – BMI≥40 kg/m2 or BMI≥35 kg/m2 in the presence of obesity related co-morbidities [21]. Patient and surgeon determined the choice of surgical procedure. The surgical procedures were performed in the standard accepted fashion [22]. Twenty-two patients were studied before or 6–12 months after surgery: RYGB (n = 5); LSG (n = 12). Seven pre-operative controls with BMI≥35 and ≤ 75 kg/m2 were included. Four patients in the preoperative group, 2 patients in the RYGB group and 4 subjects in the SG group had diagnoses of diabetes mellitus. For measurements of the DA and L-DOPA mixed meal responses, a 320 kcal liquid mixed-meal challenge (Optifast, 50% carbohydrate, 35% protein, 15% fat) consumed within a 15 minute period, was administered. Venous blood (approximately 5 ml) was drawn into EDTA tubes, in the fasted state and 15, 30, and 60 min after the meal. Venous blood samples were coded such that the surgical status of patient remained unknown to the analysis laboratory until the end of the study. Plasma was separated from whole blood by centrifugation at room temperature at 500×*g*. Plasma samples were analyzed immediately for DA by HPLC-EDC or frozen at −80 °C for batched analysis of L-DOPA by ELISA.

### 2.3 Animal studies

Castrated male Yorkshire crossbred pigs were obtained at 8 weeks of age (about 16 Kgs). Castrated Yucatan miniature swine were obtained at 2–3 months of age (<30 kgs). Male Lewis rats were obtained at 8–10 weeks of age. Male Goto-Kakazaki rats were obtained at 8 weeks of age. All animals were allowed at least one-week accommodation in the husbandry facilities of the Institute of Comparative Medicine at Columbia University Medical Center. Rodents were housed under conditions of controlled humidity (55 ± 5%), temperature (23 ± 1 °C), and lighting (lights on 0600–1800 h) with access to standard laboratory rat chow and water ad libitum. Goto-Kakazaki rodents were fed NIH #31M rodent diet. The rats were handled daily to minimize nonspecific stress before the experiments began. In most experiments, it was necessary to measure blood glucose in fasting animals. For these groups, the food was removed 8 hr before glucose levels were tested. All animal studies conducted following protocol approval and according to the guidelines of the Columbia University Medical Center Institutional Animal Care and Use Committee and the NIH Guide for the Care and Use of Laboratory Animals.

### 2.4 Cell lines and pancreatic islets source

INS-1E cells were obtained from AddexBio, Inc. and cultured according to their instructions. Purified cadaveric human islets were obtained from the Integrated Islet Distribution Program (IIDP) funded by the National Institute of Diabetes and Digestive and Kidney Diseases (NIDDK). Neonatal Yorkshire porcine islets were prepared at the Alberta Diabetes Institute of the University of Alberta as previously described [23] and shipped overnight to the laboratory. Lewis Rats islets were prepared as previously described [24].

### 2.5 Mixed meal and oral glucose tolerance testing

Eight hour fasted rats were anesthetized (5% for induction and 1.5–1.0% for maintenance, isoflurane in 90% O2 at 4.0 L/minute); ocular ointment was applied, and rats were given 1.5 mL subcutaneous warmed sterile normal saline for fluid replacement and placed prone on a heated 37 °C surface throughout the procedure. Six different gavage solutions were prepared; 1) Ensure-original formula used neat from the bottle, 2) Tyros 2 reconstituted to be isocaloric with Ensure (0.24 grams Tyros 2 powder per ml water), 3) Tyros 2 supplemented with tyrosine and phenylalanine (both at 2.5 mg/ml), 4) 50% Dextrose solution, 5) 50% Dextrose with tyrosine at 12 mg/ml and 6) Tyros 2 reconstituted and supplemented with 20 mg/ml stable isotope labeled L-tyrosine (13C9,99%, 15N 99%). Following induction of anesthesia, baseline samples of blood were drawn and whole blood glucose concentration measurements were performed with an Alphatrak glucometer. Rodents were then gavaged at 4 uL/gram body weight. Venous blood was sampled (about 300 μL) from the lateral tail vein at 0, 15, 30, 45 and 90 min following gavage. Whole blood was transferred into EDTA microcentrifuge tubes and maintained in ice. Tubes were centrifuged at 500×*g* for 15 minutes, and plasma was collected and analyzed immediately for insulin and or monoamine content or stored at −80 °C.

### 2.6 Insulin,GLP-1 and L-DOPA ELISA measurements

Measurements of Insulin, GLP-1, and L-DOPA in plasma samples were performed by ELISA following the manufacturer's instructions. Absorbance measurements were performed using a Biotek Synergy 2 plate reader.

### 2.7 Extraction of tissue monoamines

Approximately forty-five minutes following gavage, anesthetized Lewis rats were euthanized by CO_2_ inhalation. The rodent's abdomen was injected with about 50 ml of 4 °C normal saline and the upper GI tract from the esophagus to the ileocecal junction (including the spleen and pancreas) harvested en bloc and placed in chilled saline. Proceeding stepwise, first, the stomach was divided from the block, and washed in chilled saline to remove residual contents. Tissue was sampled (approximately 10 mm^2^) from glandular antrum blotted dry and placed in pre-weighed tubes (2 ml capacity containing 1 ml of 0.2 M perchloric acid, 0.1 mM EDTA and 1.5 mm Zirconium beads). These steps were repeated along the GI tract, with samples taken at 1 cm intervals. The ligament of Treitz was used to demarcate the duodenum (first 2 cm from the stomach) from the jejunum (at about one cm from the ligament) and the ileum (preceding two cm from ileocecal junction). In those experiments, using Tyros supplemented with stable isotope labeled L-tyrosine, only pancreas or brain tissue was harvested at the indicated time. Tissue was homogenized for 2 min at 4 °C using a Mini-Beadbeater-16 instrument. Homogenates were maintained an additional 30 minutes on ice, then spun at 18,000×*g* for 15 min at 4 °C. The supernatants were harvested and adjusted to pH 3.0 with 1M sodium acetate. The cleared homogenates were then filtered through 0.2 μm PTFE syringe filters for subsequent analysis by high-performance liquid chromatography-electrochemical detection (HPLC-ECD) or liquid chromatography electrospray ionization tandem mass spectroscopy (LC-ESI-MS/MS).

### 2.8 Measurements of monoamines by HPLC-ECD

For measurements of DA in patient samples, a solid phase extraction technique was used to prepare plasma samples for high-performance liquid chromatography with electrochemical detection (HPLC-ECD). One ml of patient serum was mixed with one ml of 1.0 M Tris buffer, pH 8.5 and approximately 30 mg of activated, basic, Brockmann type 1 aluminum oxide. The slurry was rotary mixed for 30 minutes at 10 rpm. The alumina oxide was allowed to completely settle, the supernatant removed and replaced with 2.0 ml of Milli Q water. This was repeated for three washes. The alumina oxide was transferred to 0.2 μm nylon Microspin centrifuge filters. The alumina oxide was spin-dried for 3 minutes at 2700×*g*. Bound monoamines were eluted by centrifugation in 0.2 M perchloric acid (PCA), 0.1 mM EDTA for analysis by HPLC-ECD. For measurements of L-DOPA and DA in rodent serum samples, solid phase extraction was not feasible. Instead, fresh plasma was precipitated with 10% PCA for 30 min on ice, spun at 18,000×*g* and the supernatants analyzed by HPLC-ECD. Samples were transferred to amber vials and placed in the autosampler. HPLC was performed as follows; 10 μl of sample or standard was injected into a high-pressure liquid chromatography with an electrochemical detector using Eicompak SC-50DS (3.0 ID x 150 mm) column prefaced with 3 mm ID x 4 mm AC-ODS guard column. The flow rate was 500 uL/minute with a column temperature set to 25 °C. The mobile phase was 20% Methanol (LC-MS grade), 80% 0.08 M Citrate-Acetate pH 3.5 buffer with 205 gms/L Sodium Octane Sulfonate and 4 mg/L EDTA-2Na. The analytes were eluted isocratically and detected with a carbon graphite electrode (WE-3G) with an applied potential of 750 mV vs Ag/AgCl. Time versus peak current data was collected and analyzed using the Eicom Envision EPC-700 chromatography software. Prior to and following each sample run, authentic standards were run at 100 pg/μl, 10 pg/μl, 1 pg μl and 0.1 pg/μl to create a calibration curve. These standards were norepinephrine, L-DOPA, DOPAC, DA, 5-HIAA, isoproterenol, HVA, 3-MT, and 5 HT. The detection limits for L DOPA and DA in plasma under these conditions were around 30 fmoles and 20 fmoles, respectively. An L-DOPA ELISA was used in confirmatory studies during methods development.

### 2.9 Positron emission tomography study of D2R occupancy in swine pancreata

The night before and the morning of the study, animals were food restricted but allowed free access to water. In the indicated experiments, 18 mg/kg AMPT and 5 mg/kg Sodium Bicarbonate were administered admixed into each of the three daily meals for two days prior to the PET scan. Alternatively, in some experiments, Haloperidol at 1 mg/kg was administered 60 minutes before the start of the PET scan. Swine were sedated with an intramuscular injection of 3–5 mg/kg of tiletamine–zolazepam combination followed by induction with propofol (2 mg/kg). Once the loss of jaw tone and palpebral reflex was observed, the pigs were intubated and maintained on isofluorane at 3–4% in O_2_. Swine were catheterized for venous access on both ears. For transport to the PET suite, isofluorane was withdrawn and additional sedation was provided via intermittent boluses of propofol (1 mg/kg) via a normal saline i.v. drip. Upon arrival, swine were returned to isofluorane inhalation anesthesia at 1.5–3% in O_2_. Body temperature was monitored by a rectal temperature probe and maintained at ∼38 °C by a heating pad. Swine were placed supine and head in the PET scanner to allow venous access once the swine's abdomen was in the correct field of view. PET scans were performed on a Biograph mCT PET/CT camera with a reconstructed spatial resolution of approximately 4 mm. Data were acquired for 180 min. A low-dose abdominal computerized tomography (CT) scan, guided by the CARE Dose4D program (120 kV, 50–350 mA), was performed at the beginning of each session for the collection of attenuation mapping and anatomical data. Dynamic PET measurements were acquired in 3D mode over an axial field of that covered 22 cm beginning near the xiphoid process and extending caudally over the abdomen. List mode-acquired PET data were reconstructed into 24 (3 h) dynamic frames with increasing frame duration using an iterative ordered subset expectation maximization algorithm (4 mm FWHM, Hann filter, two iterations/24 subsets) with CT-based attenuation, time-of-flight scatter, random, scanner dead time, detector normalization, and radioactive decay corrections as provided by the manufacturer's software. The final reconstructed images had a matrix size of 168 × 168 × 74 and a voxel size of 4 × 4 × 3 mm. 18F-Fallypride in a sterile saline vehicle was obtained from PETNET solutions with the typical end-of-synthesis specific activity of 200 MBq/nmol. Fasting blood glucose measurements performed at this revealed an average BG of 68 ± 4 mg/dL 18F-Fallypride (mean dose of 204 ± 13 MBq) was administered as a bolus over 2–3 min immediately after the initiation of the scan. The mean specific activity of 18F-Fallypride at the time of injection was 22 ± 15 MBq nmol−1. There was no statistically significant difference in injected mass at baseline and following challenge. A normal saline flush was performed after the injection of tracer for baseline scans. In the indicated experiments, the saline flush was substituted with 50% Dextrose at 1 ml/kg. The glucose challenge was performed via the i.v. route, rather than p.o, because the animals were anesthetized, intubated and the delivery of glucose to the endocrine pancreas was more precise and predictable within the time-frame of optimal tracer delivery to the pancreas using the i.v. route. The glucose challenge scans were performed within 4–6 weeks following the baseline scan.

#### 2.9.1 Quantification of PET data

From the reconstructed PET and CT data, a fused image was provided by the Syngo software. The frame by frame reconstructed PET data were analyzed with PMOD software. Volumes of interest were manually drawn on summed images to generate the time activity curves for pancreas (Body and Tail) and spleen. Radioactivity in all VOIs was calculated as the mean radioactivity concentration (KBq/mL). To generate standardized uptake values (SUVs) the VOI activities were normalized to the injected dose and corrected for animal weight. Next, using the spleen as reference tissue input, 18F-Fallypride D2R binding potential, BPND, which is linearly proportional to the D2R density (Bavail), was calculated for the combined body and tail of the pancreas using the reference tissue model, MRTM2 in PMOD as previously described [25]. MRTM2 utilizes kinetic data to estimate BPND but BPND itself represents the equilibrium ratio of specifically bound to nondisplaceable tracer (free plus non-specifically bound, assumed to be the same in all tissues at equilibrium). The effect of radiolabeled metabolites on the percent change following glucose or pharmacological challenges can be approximated in the equilibrium setting. In brain, the fractional change is

(Cs(base)−Cs(post))/Cs(base)

where Cs(base) and Cs(post) are the equilibrium concentrations of specifically bound (to D2R) tracer at baseline and after the challenge. Under the assumptions that i) metabolism of the radiotracer is unaffected by the challenge and ii) the equilibrium nondisplaceable tracer concentrations are the same in pancreas and spleen, the fractional difference in BPND across conditions in pancreas is

(Cs(base)−Cs(post))/(Cs(base)+Cm(pan)+Cm(spl))

where Cm (pan) and Cm (spl) are the radiolabeled metabolite concentrations in pancreas and spleen. To the extent that the radiolabeled metabolites accumulate similarly over time in both tissues, the percent change in BPND will approximate the percent change in specific binding of the tracer. The early phases (<10 minutes) of the tracer TACs reflect VOIs with minimal 18F-Fallypride metabolites contamination [26].

### 2.10 In vitro isotopomer labeling studies

Human islets were cultured for 24–36 hrs following receipts in non-adherent T175 flasks at 37 °C. The transport media was replenished daily with fresh CMRL-1066 medium, supplemented with 1.5% human albumin, 0.1% insulin-transferrin-selenium (ITS), Penn/Strep, 5 ml of 1M HEPES, and 14.5 ml of 7.5% Sodium Bicarbonate. INS-1E cells were cultured in RPMI 1640 medium supplemented with HEPES, glutamine, sodium pyruvate and -mercaptoethanol as previously described [8]. A tyrosine free modification of this media was prepared and supplemented with 5% BSA and 5% dialyzed fetal calf serum. Islets (400 IEQ/well) or INS1E cells (confluent 100 cm^2^ flasks) were cultured overnight in the tyrosine-free media. Cultures (viability > 90% by trypan blue exclusion) were then supplemented with 2 mM L-tyrosine (Ring-13C6, 99%) or 10 mM L-DOPA (Ring-D3, 98%). Where indicated cultures were also supplemented with 10 uM pargyline, 10 uM Moclobemide and 10 uM GBR 12909 to inhibit catabolism of DA. At the indicated times, culture supernatants were harvested and cell/islet perchloric acid extracts prepared. Supernatants (5 ml) were processed by solid phase extraction as described above. PCA cell extracts were cleared by centrifugation. Samples were then purified by HPLC as described above and sample fractions corresponding to the elution times of L-DOPA and DA collected and frozen at-80 °C for later LC-ESI-MS/MS analysis.

#### 2.10.1 LC-ESI-MS/MS

Dopamine isotopic enrichment was determined as native dopamine and as the n-propyl derivative [27] (diester, amide) by LC-MS/MS (Shimazu UFLCXR, QTRAP 6500) by negative electrospray ionization. Three parent-daughter ion pairs were monitored to confirm peak identification as dopamine (154/137, 154/119, and 154/91), or as its n-propyl derivative (322/137, 322/210, and 322/266). A gradient elution Solvent A: aqueous ammonium acetate 12 mM, Solvent B: acetonitrile) using a C8 reverse phase HPLC column (on a Phenomenex Luna 5u C8, 100A, 150 × 4.6 mm, 5 microns) was used for dopamine (gradient: 5–20% solvent B) and the dopamine n-propyl derivative (gradient: 50–70% solvent B). Isotopic enrichments were determined by monitoring parent-daughter ion pairs of dopamine (and the n-propyl derivative) formed from the labeled precursor: dopamine m+3 from [2, 5, 6-D3] L-Dopa, dopamine m+6 from ring [13C3]tyrosine, and dopamine m+9 from [13C9, 15N]tyrosine. Tyrosine isotopic enrichment was determined following derivatization as the n-trifluoracetyl-n-butyl ester [28]by Chemical ionization GC-MS (Agilent: HP-1 column, G1530A GC, and 5975C MS) using isobutane as the reagent gas, He gas at a flow rate of 1 ml/min was the carrier, and a temperature program ramped from 100 to 200 °C.

### 2.11 Immunohistochemistry

Rodent foregut and pancreas or swine pancreas tissue were fixed in 4% paraformaldehyde in 0.2M phosphate buffer and paraffin embedded using standard methods. Sections were cut at 5 μm thickness and antigen retrieval was performed in 10 mM citric acid, 0.05% Tween 20, pH 6.0 at 96 °C for 15 min. We used Anti tyrosine hydroxylase, anti-aromatic amino acid decarboxylase, anti-VMAT1, anti-chromogranin A, Anti-insulin [29] and Anti-Dopamine 2 like receptor [30] antibodies at dilutions of 1:500, 1:500, 1:1000, 1:2,000, 1:2,000 and 1:500, respectively. We visualized the immune complexes with FITC- or CY3-conjugated secondary antibodies. DAPI staining was used as a nuclear marker. Stained sections were imaged on an epifluorescent microscope equipped with an Infinity 2 monochrome CCD camera. Images were processed with the Infinity Analyze software.

### 2.12 Assessment of VMAT2 protein expression by western blot assay

Lewis rats' tissues, including purified islets, were homogenized in 10 mmol/l Tris homogenization buffer (pH 7.4) with Halt protease inhibitors cocktail added as per the manufacturer's recommendations. The samples were centrifuged at 14,000×*g* for 30 min at 4 °C and the supernatant collected for western blot assay. Protein concentrations of the supernatants were determined by the BCA method and 15 μg protein of each sample was loaded onto the 10–20% SDS gel, separated by electrophoresis in a tris-glycine buffer and transferred to nitrocellulose (NC) membrane. The membranes were blocked in blocking buffer (5% nonfat milk) for 2 h at room temperature and then were incubated with rabbit anti-VMAT2 polyclonal IgG AB1598P overnight at 4 °C. After being washed three times for 10 min with washing solution, the membranes were incubated with goat anti-rabbit IgG-HRP and immunoreactive bands were visualized by an ECL Western blotting detection kit using a FluorChem M System. The images were analyzed using Alphaview software.

### 2.13 Live cell microscopy

Pig islets were cultured in complete Ham's F10 medium for 2–3 days at 37 °C, 5% CO_2_ and 95% air in non-treated culture flasks. Islets were then washed in Hanks balanced salt solution and treated with 0.25% Trypsin, 0.1% EDTA in HBSS to effect islet dispersal to single cells. The suspensions were washed and then filtered through sterile nylon 100 um mesh filter. Cells were transferred to RPMI1640 without Phenol Red but supplemented with 10% glutamine, 10 mM HEPES, 1% penicillin/streptomycin and 1% FBS and 5% BSA and plated at 2mls/well in poly-lysine coated 0.17 mm thick glass coverslip chamber slides (Lab-Tek II) for overnight culture. Adherent islet cell cultures were treated with 10 uM DnsykD-1 probe at 1000 x the Kd. Some cultures received 1 mM Haloperidol in addition to DnsykD-1. Cell cultures were maintained at 37 C in 5% CO2 and 95% air, until imaged at 5, 15, 30, and 45 minutes after the addition of DnsykD-1. Cells were imaged with Zeiss Axiovert 135 inverted microscope equipped with a heated platform (Warner, CSH-1), a single channel temperature controller (Warner, TC-324C), a 385 nm LED source (Thorlabs, M385LP1) and 400 nm long pass emission filter. Images were captured with a Lumenera Infinity 2 monochrome CCD camera. Images were processed with the Infinity Analyze software.

### 2.14 Analysis of D2R and VMAT2 expression by RT-PCR

Total RNA from INS-1 E cell line, purified pancreatic islets, purified acinar tissue, and rat brain were isolated using the RNeasy Lipid Tissue Mini Kit. cDNA from porcine tissues was generated using the VILO cDNA synthesis kit (from 1 μg of the isolated total RNA). Retro Transcription of total RNA from rat tissues and INS-1 E cells has been instead done using the 3′ RACE System for Rapid Amplification of cDNA Ends, according to the instructions provided by the manufacturer (from 4 μg of the isolated total RNA). To perform PCR assays the amount of cDNA obtained retro-transcribing 30 ng total RNA was used in each reaction for pig tissues, 100 ng of total RNA for rat brain and INS-1 E, and 250 ng for purified rat islets RNA. AccuPrime™ Pfx SuperMix was used for semiquantitative PCR assays at the conditions recommended by the manufacturer; varying the annealing temperature and the extension time on the basis of the couple of primers used. All the primers (Table S2) were synthesized by Invitrogen Custom Primers except for the AUAP (Abridged Universal Amplification Primer) that is included in the 3′ RACE System. The PCR products were run on a 2% agarose gel in 1X TAE buffer, stained with a 3X GelRed™ Nucleic Acid Gel Stain solution, imagined and quantified by densitometry using Alphaview software.

### 2.15 Data analysis and statistics

Descriptive and comparative statistic including the arithmetic means, standard errors of the mean (SEM), Mann Whitney U test, Students t-testing and parametric and nonparametric repeated measure ANOVA were calculated using Medcalc software. Data are presented as the Mean ± 1 SEM. The significance of comparisons (p values) of statistical means was calculated using the 2-tailed Student's t-test unless indicated otherwise. P values < .05 were considered statistically significant. The significance of differences in tissue monoamine content kinetics was calculated using Friedman's nonparametric repeated measure ANOVA or in the case of human L-DOPA and DA excursions, a standard parametric repeated measure ANOVA was applied

## 3 Results

### 3.1 GI tissue content of L-DOPA and DA is responsive to the tyrosine content of the mixed meal stimulus

Using immunohistochemistry with previously validated antibodies, we studied the expression of TH and AADC in the foregut (i.e. stomach and small bowel) of the rat (Supplemental Materials, Figure S1). These enzymes are responsible for the conversion of tyrosine to L-DOPA and the conversion of L-DOPA to DA, respectively. Both TH and AADC were expressed in cells of the gastric mucosa, occasional enteroendocrine cells in the intestinal mucosa and throughout the lamina propria (or illeal submucosa) of the small bowel, confirming and extending previous results [5], [31]. We also analyzed the expression of vesicular monoamine transporter type 2 gene (VMAT2) in rat tissues, including pancreatic islets (purified from the pancreas of Lewis rats) and total brain, either at level of protein (by western blot) and as accumulation of specific RNA (by semi-quantitative RT-PCR) (Supplemental Materials, Figure S2). We confirmed the expression of the VMAT2 gene in rat pancreatic islets as previously reported [32] and the presence of VMAT2 transcripts in INS-1 E, a rat insulinoma cell line used as a model of in vitro insulin secretion [10].

**Figure 1:**
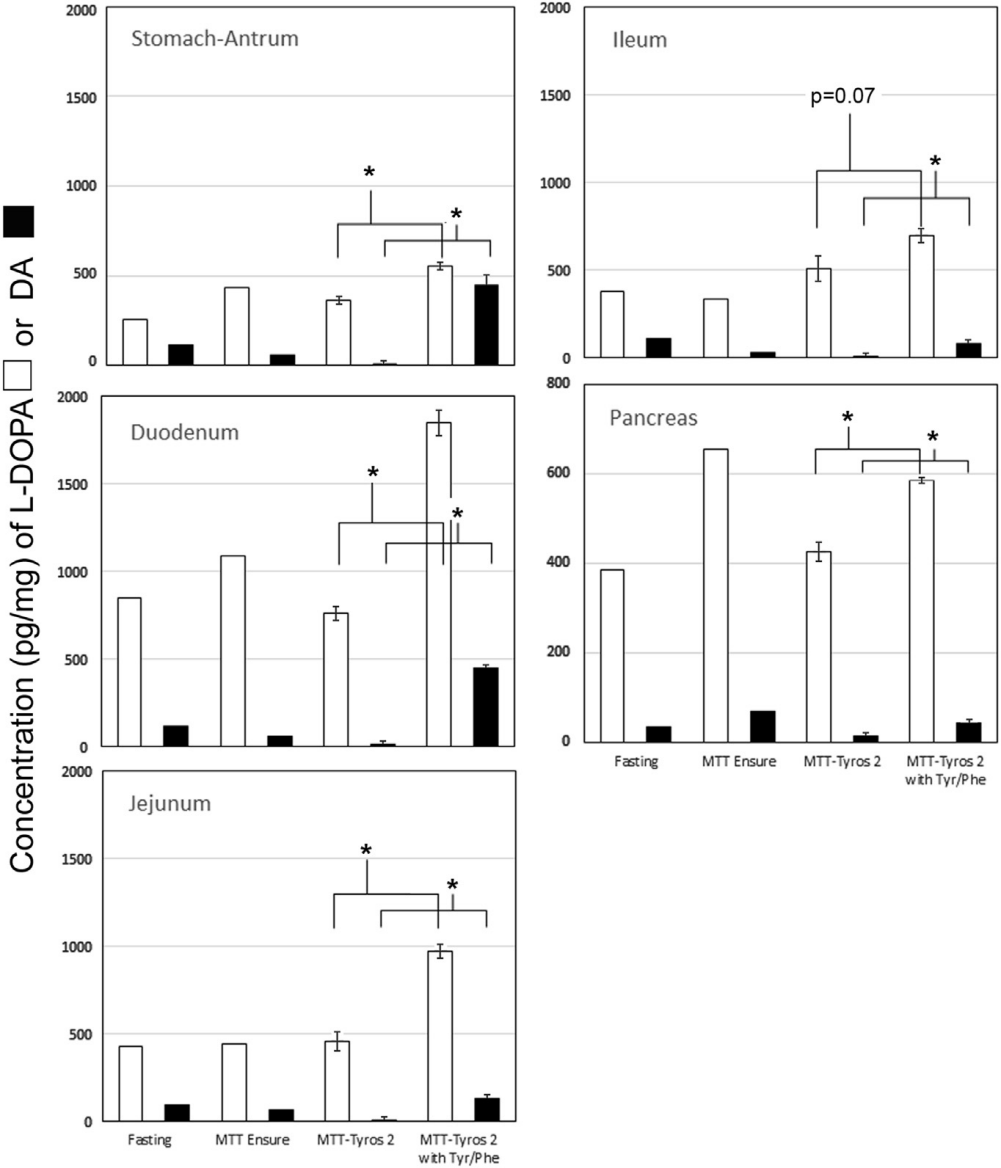
**G.I. tissue L-DOPA and DA content is responsive to the tyrosine and phenylalanine content of the mixed meal stimulus**. Male Lewis rats (n = 4 per group) were gavaged with isocaloric mixed meal formulas that differed in tyrosine (TYR) and phenylalanine (PHE) content. Forty-five minutes after gavage, the rodents were euthanized, and tissue from the gut and pancreas was harvested and analyzed for L-DOPA (white bars) and DA (black bars) content using two technical replicates. The mean wet tissue weight normalized monoamine content along the GI tract for each stimulus is displayed. A non-parametric repeated measure ANOVA (Friedman's test) was applied to the data set revealing that GI tissue L DOPA and DA content in Tyros 2 fed animals was significantly lower (p < 0.05) from the three other conditions. Likewise GI tissue L-DOPA-DA content in Tyros 2 + TYR/PHE was significantly higher (p < 0.05) from the three other conditions. The statistical significance of the differences between MMTT-Tyros and MMTT-Tyros + TYR/PHE stimulated L-DOPA and DA tissue content was performed using a two-tailed Students t-test. Error bars represent the standard error of the mean (SEM). Only the statistical significances of the measured differences between Tyros2 and Tyros2 with TYR/PHE are shown (* = p < 0.05).

Previous studies in rodents have demonstrated that the concentrations of L-DOPA and DA in the stomach, small and large intestines fall or rise during fasting and feeding, respectively [33], [34]. To investigate whether L-DOPA and DA content of the GI tissue was responsive to TYR content of a meal, we measured the concentration of L-DOPA and DA in the foregut and hindgut (i.e. stomach to ileum including pancreas). Tissues were obtained from Lewis rats in the fasting state, after a standard mixed meal (Ensure), and after an isocaloric mixed meal prepared with a TYR and PHE-free food powder (Tyros 2). Additional tissue was obtained from rats feed with Tyros 2 supplemented with TYR and PHE to the levels present in the standard mixed meal formula (Ensure). We found that the GI tissue content of DA and L DOPA (Figure 1) varied significantly depending on fasted/fed state and the type of meal challenge administered. MMTT with Ensure resulted in higher concentrations (p < 0.05) of DA in antral and duodenal tissue relative to fasting, consistent with previous studies [33]. When the mixed meal stimulus was performed with Tyros 2, all tissues studied showed significantly reduced levels of DA (p < 0.05) relative to fasted rats or rats fed Ensure. MMTT with Tyros 2 supplemented with free TYR and PHE significantly increased tissue contents of DA to levels equivalent to or higher than those following MMTT with Ensure. Non-parametric ANOVA indicated that GI tissue DA concentrations were not significantly different between fasting or Ensure-fed rats. However, Tyros 2-fed rats had significantly lower GI tissue DA content than fasting or Ensure-fed rodents (p < 0.05). GI tissue Dopamine content (expressed as pg analyte/mg wet tissue weight) of rodents given Tyros 2 supplemented with TYR and PHE was significantly higher (p < 0.5) than that measured in the other groups. Changes in L-DOPA content of GI tissue were quantitatively less than DA content changes. The L-DOPA content of GI tissue in Ensure-fed rodents was generally higher than that of fasted rodents (p < 0.05). Tyros 2 feeding reduced upper GI tissue levels of L-DOPA to those present in fasted animals (p < 0.05). Tyros 2 supplemented with TYR and PHE meal stimulus showed significantly higher levels (p < 0.05) of L-DOPA along most of the GI tract.

### 3.2 Relationship of glucose tolerance and insulin secretion to tyrosine content of the meal stimulus

**Figure 2:**
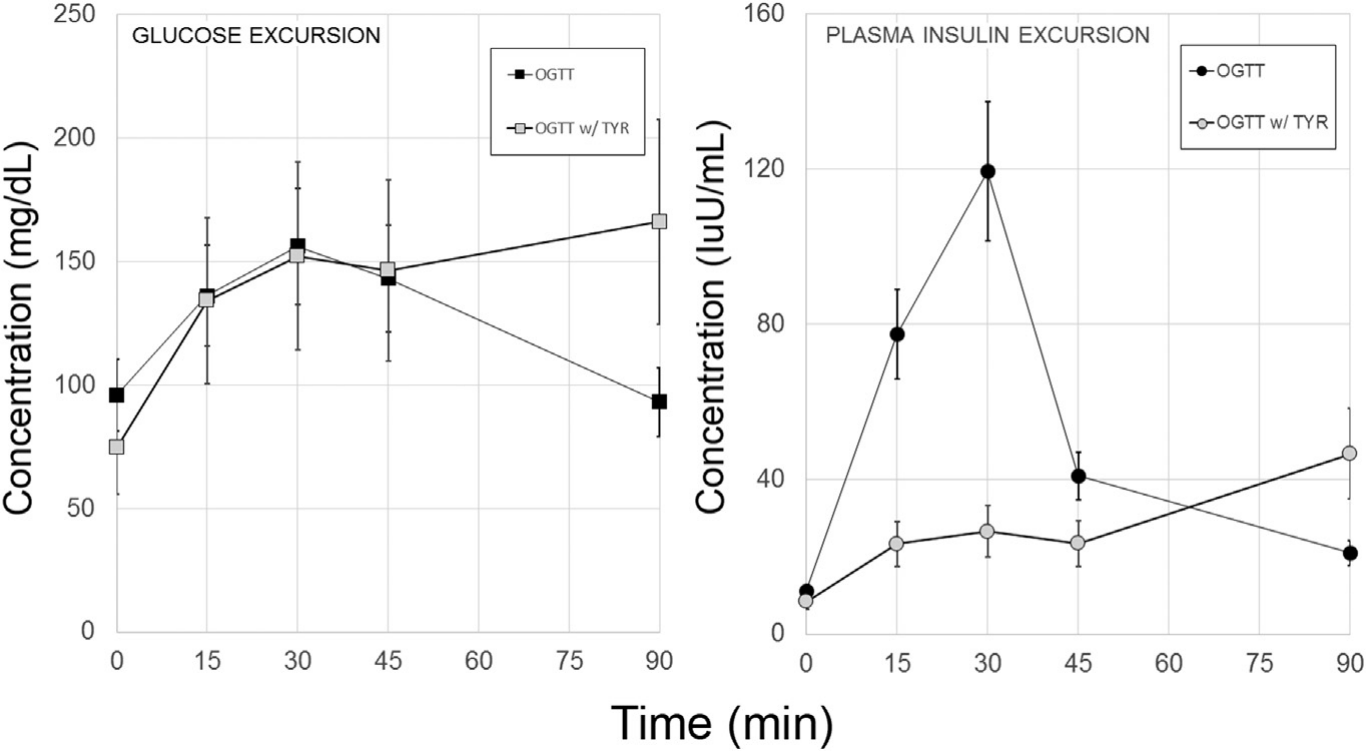
**Exogenous tyrosine affects oral glucose tolerance and insulin secretion**. Lewis rats were gavaged with a dextrose solution with or without L-tyrosine. Measurements of whole blood glucose concentrations and plasma measurements of insulin were made in the serial plasma samples. Individual plasma insulin concentrations were obtained from two technical replicates. Left panel, Time versus whole blood glucose concentration. The excursions were significantly different (p < 0.02) by a repeated measures Bonferroni-corrected ANOVA. Right panel. From the time versus insulin concentration profiles, the area-under-the-curve was calculated. The insulin excursions in the presence of glucose and tyrosine (OGTT W/TYR) were significantly smaller relative to the excursion measured with glucose alone (OGTT). Error bars represent the SEM. From a representative experiment in a series of two.

To investigate whether nutritional tyrosine might influence glucose tolerance, we performed oral glucose tolerance tests with and without added free tyrosine in Lewis rats to isolate the role of tyrosine in glucose homeostasis (Figure 2). The metrics of glucose tolerance for these experiments, including rodent weights, mean fasting glucose and insulin concentrations, mean glucose and insulin area-under-the-curve (AUC) as wells as the HOMA-IR and Matsuda indices of insulin sensitivity and the statistical significances of the observed differences are summarized in Table 1. Relative to glucose alone insulin excursions (insulin AUC) were significantly decreased when glucose and TYR was administered. A repeated measures ANOVA of the blood glucose data also revealed a significant difference in the kinetic profiles of glucose versus glucose plus TYR (p < 0.05). The presence of TYR in the oral glucose solution was associated with a significant (p < 0.005, two-tailed Students t-test)) elevation in the 90-minute blood glucose concentration relative to the oral glucose solution alone. Goto-Kakazaki rats are a well-accepted rodent model of T2D [35] that has been studied in the context of bariatric surgery [36]. We performed OGTT testing in this model (with and without TYR) and obtained similar results to our experiments in Lewis rats (Figure S3).

**Table 1:** Summary of glucose homeostasis secondary outcome measures.

Treatment	n^a^	Mean Weight (gms)	Mean Fasting Glucose (mg/dL)	Mean fasting insulin (IuU/ml)	Mean Glucose AUC (mg/dL*min)	Mean INS AUC (IuU/ml*min)	Mean HOMA-IR (pM*mM)^b^	Mean Matsuda Index^c^ (ISI composite) (IuU/ml*mg/dL)^−1^	Ii^g^
OGTT	5	408 ± 28^d^	96 ± 11	11.1 ± 3	11499 ± 859^e^	3804 ± 540	17 ± 5	4.5 ± 0.7	0.33 ± 0.22
OGTT w/TYR	6	362 ± 104	75 ± 4	8.5 ± 1	15154 ± 1262	1511 ± 334	12 ± 5	7.1 ± 1.2	0.10 ± 0.03
p Value^f^		0.41	0.42	0.29	0.047	0.004	0.29	0.18	0.0003
MMTT	7	249 ± 18	128 ± 8	50.1 ± 8	15272 ± 1245	3849 ± 1138	16 ± 2	1.61 ± 0.2	0.26 ± 0.03
Tyros 2	8	254 ± 18	103 ± 7	60.7 ± 17	12476 ± 293	7782 ± 3980	17 ± 6	1.64 ± 0.3	0.59 ± 12
P Value		0.85	0.11	0.58	0.046	0.027	0.84	0.93	0.023

a Number of male rodents per group. From one representative experiment in a series of two.

b HOMA-IR calculated as described in [37].

c Matsuda index of insulin sensitivity calculated as described in [38].

d Results are presented as mean ± standard error of the mean.

e From time versus concentration profiles, the areas-under-the-curve were calculated by the trapezoidal rule.

f p values calculated using a two-tailed Students t-test.

g Ii, Insulinogenic index ((min*IuU/ml)/(min*mg/dL)) calculated as described in [39].

### 3.3 Relationship of glucose tolerance and insulin secretion to the tyrosine content of the mixed meal stimulus

**Figure 3:**
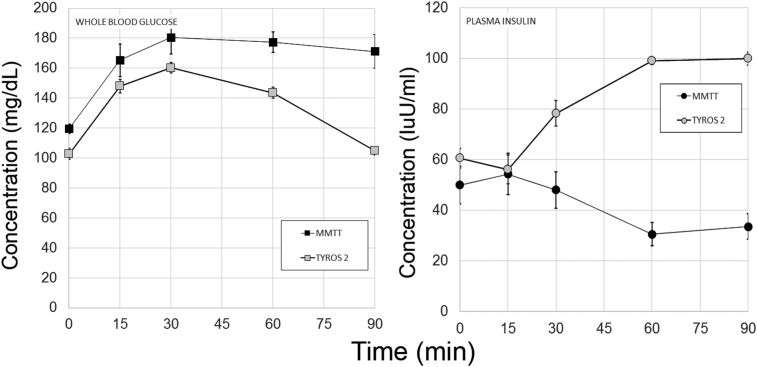
**Tyrosine content of the meal stimulus affects glucose tolerance and plasma insulin excursions**. Male Lewis rats were gavaged with Ensure or isocaloric TYR/PHE free Tyros 2, followed by serial blood sampling over a 90 min. period. Measurements of whole blood glucose concentrations (left panel) and plasma measurements of insulin (right panel) were made in the serial samples. Error bars represent the SEM. Plasma insulin concentrations obtained from two technical replicates. From a representative experiment in a series of two.

Oral glucose tolerance testing demonstrated that glucose excursion were significantly higher in the presence of exogenous oral tyrosine. Aside from demonstrating a possible mechanism for the defense against hypoglycemia, creating glucose intolerance was not of particular preclinical interest. We returned to the mixed meal tolerance testing we described for the measurements of gut tissue L-DOPA and DA content experiments. To investigate whether differences in mixed meal stimulus tyrosine content affected glucose homeostasis, we performed MMTT in normoglycemic Lewis rats using Ensure and an isocaloric dose of Tyros 2 (i.e. PHE/TYR depleted liquid meal formula). Based on the previous experiments, we posited that an MMTT with Tyros 2 would result in lower circulating glucose concentrations and larger insulin excursions (Figure 3). The metrics of glucose tolerance for these experiments, including rodent weights, mean fasting glucose and insulin concentrations, mean glucose and insulin area-under-the-curve (AUC) as wells as the HOMA-IR and Matsuda indices of insulin sensitivity, the Insulinogenic index and the statistical significances of the observed differences are summarized in Table 1. As predicted, the plasma insulin excursions were significantly increased when Tyros 2 was substituted for the Ensure MMTT stimulus. Comparison of the blood glucose concentration versus time profiles obtained with Ensure versus Tyros 2, showed the Tyros 2 AUC was significantly lower than the Ensure MMTT AUC. We also examined GLP-1 excursions in this model. We did not find any significant differences (p = 0.54) in the GLP-1 excursions in response to Ensure (n = 5, AUC = 1091 ± 57 pM*min) compared to Tyros 2 (n = 6, AUC = 995 ± 130 pM*min) as determined by the areas-under-the curve.

### 3.4 L-DOPA and DA excursions following a tyrosine depleted meal stimulus or bariatric surgery are smaller relative to controls

**Figure 4:**
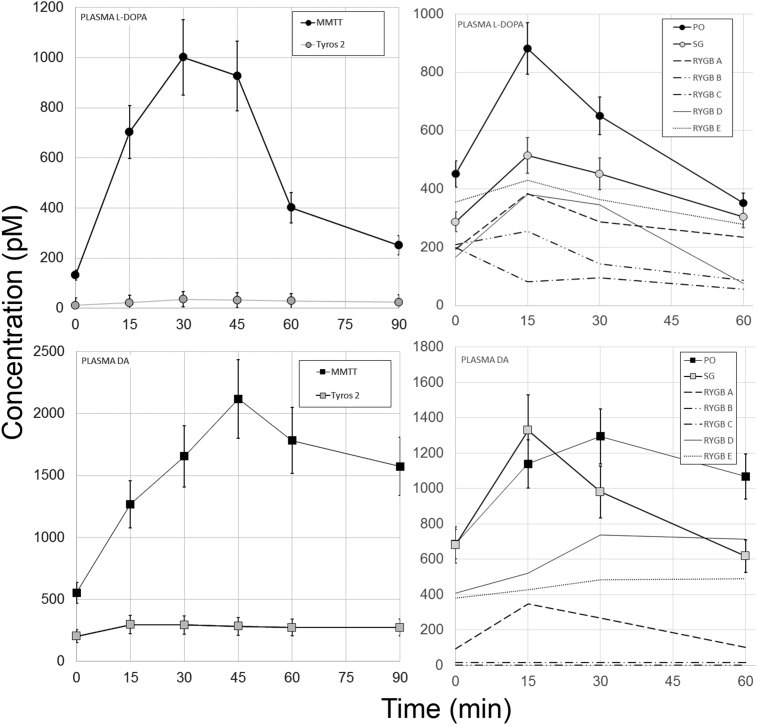
**Plasma L-DOPA and DA excursions in response to Mixed Meal Testing in a population of Lewis rats and Bariatric Surgery patients**. Left panels. Male Lewis rats were gavaged with Ensure or isocaloric TYR/PHE free Tyros 2, followed by serial blood sampling over a 90 min. period. Measurements of whole blood glucose concentrations (left panel) and plasma measurements of L-DOPA and DA (Left panel) were made by HPLC-ECD in the serial samples. Plasma monoamine concentrations obtained from two technical replicates. Right panels. Preoperative control patients (PO), patients with vertical sleeve gastrectomy (SG) and patients with Roux-en-Y-gastric bypass (RYGB) were given a mixed meal tolerance test, followed by serial blood sampling over a 60 minute period. DA measurements were performed by HPLC-ECD. L-DOPA measurements were performed by ELISA. Measurements of plasma L-DOPA and dopamine obtained from two technical replicates. Error bars are the SEM. The individual profiles of each RYGB patient are presented rather than the mean excursions shown for the PO and SG populations.

There are significant post-prandial excursions of plasma DA and L-DOPA following a mixed meal challenge in both humans [4], [7] and rodents [8]. Based on our studies of rodent GI tissue L-DOPA and DA content following various mixed meal challenges (Figure 1), we hypothesized that plasma excursions of L-DOPA and DA would vary according to the meal stimulus (Figure 4, **Left panels**). As predicted, the plasma excursions, as reflected by the AUCs, of both L-DOPA and DA were significantly smaller (Table 2) when the TYR/PHE depleted Tyros 2 meal stimulus was substituted for the standard mixed meal challenge Ensure.

**Table 2:** Summary of L-DOPA and DA excursions following MMTT.

Group	n	AUC L-DOPA (nM*min)	SEM	AUC DA (nM*min)	SEM
MMTT	9	53.3^a^	2.6	143.5	4.8
Tyros 2	8	2.4	0.8	28.0	5.1
p-value		0.04		0.03	
PO	7	32.6	6.8	55.3	12.0
SG	10	24.0	4.0	50.6	17.4
RYGB	5	13.3	2.9	10.6	5.2
p-value					
PO vs SG		0.03		0.65	
PO vs RYGB		0.014		0.005	

a Plasma monoamine concentrations determined by HPLC with ECD detection from two technical replicates.

Others and we demonstrated that foregut tissue (i.e. stomach and duodenum) contain significant amounts of TH and AADC, two enzymes responsible for the conversion of TYR to L-DOPA and DA. Bariatric surgeries such as sleeve gastrectomy (SG) and Roux-en-Y gastric bypass (RYGB) exclude the nutrient path from much of these tissues containing TH and AADC. We hypothesized that exclusion of nutrients containing TYR from these tissues might result in changes in the L-DOPA and DA excursions normally seen after MMTT and similar to those observed when an isocaloric, but TYR depleted liquid meal was substituted. To test this idea, we performed a cross-sectional study of plasma L-DOPA and DA concentrations following a MMTT in a group of subjects recruited from the Weight Control Center at Columbia University Medical Center. The twenty-two bariatric surgery subjects studied were 7 obese pre-operative patients (PO), 10 patients after laparoscopic sleeve gastrectomy (SG), and five patients after RYGB (subject characteristics presented in Table 3). Serial measurements of plasma L-DOPA and DA were obtained during the MMTT. The kinetics of elaboration of L-DOPA and DA – as reflected in serial serum assays - were significantly different among the patient groups (Figure 4, **right panels**). RYGB surgery patients had significantly smaller plasma L-DOPA and DA excursions than the PO subjects as reflected in the area-under-the-curves (AUCs) comparisons (Table 2). There was a trend toward lower plasma DA AUC concentrations in the SG population relative to the PO subjects that did not reach statistical significance. However, if only the mean 60 min serum DA concentration was considered, there was a significant difference in DA levels (p < 0.05, two-tailed t-test) between the SG patients and PREOP patients. The concentration vs. time profile of plasma L-DOPA in the SG patients was significantly different from that of the PREOP and RYGB patients (p < 0.05 by a non-parametric Friedman test). Lastly, the L-DOPA and DA excursions measured in RYGB patients where most similar to those excursions observed when a TYR depleted liquid meal was substituted for the normal Ensure stimulus (Figure 4 **comparison of right and left panels**).

**Table 3:** Bariatric Study population characteristics.

Procedure	M/F	BMI (kg/m^2^)	Weight loss (%)	Preoperative DM/total	Time after procedure (yrs)
Preoperative (PO)	3/4	45±4^a^	0	4/7	0
Sleeve Gastrectomy (SG)	4/6	37 ± 1	23 ± 4	4/10	1.5
Roux-en-Y Gastric Bypass (RYGB)	3/2	30 ± 1	32 ± 3	2/5	2.7

a Demographic measures presented as Mean ± S.E.M.

### 3.5 Determining the metabolic fate of oral tyrosine

Our rodent glucose tolerance experiments suggested that TYR might be an important modulator of glucose homeostasis. To understand better the metabolism of TYR and its relationship to glucose homeostasis, we traced the transformation of oral tyrosine into dopamine stored in beta cells. We showed that human cadaveric islets, and the rodent INS1E β-cell, transformed isotopically-labeled (deuterated) L-DOPA and [13C] labeled tyrosine into dopamine in vitro. Cells and islets were incubated in tyrosine-free media and the cultures treated with either labeled L-DOPA or L-tyrosine. After the indicated incubation period, catecholamines were purified from the culture supernatants or cell extracts by high-performance liquid chromatography and analyzed by liquid chromatography-electrospray ionization tandem mass spectroscopy. Percent isotopic enrichments for L-DOPA and DA were calculated for each condition (Figure S4). Both human islets and INS1E cells metabolized L-DOPA into DA as reflected by the enrichment stable isotope-labeled DA or L-DOPA, extending and confirming previous studies [40]. Human islets metabolized both L-DOPA and TYR into DA. The conversion of TYR and L-DOPA into DA was enhanced in the presence of inhibitors of DAT (GBR12909) and MAO (pargyline and moclobemide). MAO catalyzes the oxidation of DA into 3,4 dihydroxyphenylacetic acid.

Next, we repeated the Tyros 2 MMTT rodent experiments, substituting tyrosine with stable isotope-labeled tyrosine. At the indicated interval following gavage, the rodents were euthanized and the pancreas harvested for measurements of isotopic enrichment of DA (Table 4). We found that oral tyrosine [13C9,15N] became detectable in plasma as early as 60 minutes after gavage of rodents with Tyros 2 supplemented with tyrosine [13C9,15N]. Pancreatic dopamine [13C8,15N], a metabolic derivative of tyrosine [13C9,15N], was estimated to be enriched relative to baseline to approximately 3% at 60 and 90 minutes and to over 4%, at 120 minutes, respectively, following gavage. For purposes of comparison and as a control, we measured the isotopic enrichment of dopamine in whole brain tissue at 120 minutes following gavage. The enrichment of dopamine [13C8,15N] was approximately 2% in brain tissue and was significantly lower than that found in the pancreas.

**Table 4:** Oral Tyrosine is transformed into pancreatic DA.

Time (minutes)^a^	Plasma [13C9,15N] Tyrosine		Pancreas [13C8,15N] Dopamine		Brain [13C8,15N] Dopamine	
Percent isotopic enrichment (mean ± s.e.m)						
0^b^	<0.01		<0.01		<0.1	
60	5.2 ± 0.2	p < 0.003^c^	3.1 ± 1.8	p < 0.001^c^	n.d.	
90	1.8 ± 0.3		3.8 ± 2.1		n.d.	
120	1.1 ± 0.5		4.4 ± 1.4		2.1 ± 0.6	p < 0.02^c^

a Lewis rats were gavaged with Tyros 2 supplemented with 20 mg/ml stable isotope labeled L-tyrosine (13C9,15N). At the indicated times blood samples were drawn and the rodents were euthanized and the pancreas and/or whole brain tissue harvested. Plasma tyrosine and tissue dopamine isotopic enrichment for each sample (triplicate determinations) was measured for each time point by LC-ESI-MS/MS. Plasma and tissue enrichments are the average of enrichment at t = 0 (n = 2), t = 60 (n = 2), t = 90 (n = 2), and t = 120 min (n = 2). n.d. not done.

b The baseline t = 0 time isotopic enrichment of [13C9,15N] Tyrosine and [13C8, 15N] dopamine was measured and found to below the detection threshold of the instrument. It is estimated to be the product of the natural isotopic abundances of 13C and 15N.

c The 60, 90, and 120 min plasma isotopic enrichment values of [13C9,15N] Tyrosine or pancreatic dopamine isotopic enrichment values were pooled and compared to the measured baseline abundance of [13C9,15N] Tyrosine or [13C8, 15N] dopamine using a Mann–Whitney test. Results from a single experiment.

### 3.6 PET imaging of pancreatic D2R occupancy following in vivo islet glucose-stimulated DA secretion

One element of the proposed glucose homeostasis regulatory circuit suggests that glucose challenge results in the release of DA from beta cell vesicular stores and subsequent inhibition of GSIS via binding to D2R expressed by beta cells. To assess whether β-cell stores of dopamine are released and bind to β-cell dopamine receptors in situ, we applied an experimental paradigm used to measure dopamine receptor occupancy in the CNS [41]. Briefly, in the CNS, measurement of D2R occupancy (by DA) is performed by competition with the moderate affinity D2R PET ligand, 18F-Fallypride [42]. The human CNS imaged with 18F-Fallypride at baseline and then a second time, following amphetamine challenge, demonstrates a significant loss of bound PET ligand in the striatum [42]. Administration of amphetamine or cocaine is known to increase extracellular striatal DA concentrations [43], and the loss of bound PET ligand signal is due to endogenous DA competing with 18F-Fallypride for dopamine receptor occupancy. Similarly, the drug alpha-methyl-para-tyrosine (AMPT), an inhibitor of tyrosine hydroxylase and dopamine synthesis, when administered to subjects prior to PET scan with certain D2R PET ligands, reveals higher PET ligand binding as a result of the loss of competition with endogenous DA [44].

We used swine as our model system because of the physiological similarity between human and swine islets. In swine the pancreas volume of interest approaches that found in humans and is about 100 x larger than that of rats, allowing for accurate quantification of the PET ligand signal. We quantified D2R occupancy in swine pancreata using the D2R PET ligand 18F-Fallypride at baseline (all fasting blood glucose concentrations were ≤68 mg/dL), and following i.v. glucose challenge (0.5 gm/Kg body weight) using Binding Potential (BPND) as the outcome measure. In the brain, BPND is directly related to the receptor density in the observed tissue [45]. The blood–brain barrier prevents radiolabeled metabolic byproducts of the breakdown of 18F-Fallypride from entering the brain, but in tissues external to the brain, radiolabeled metabolites are likely to contribute to the signal. Still, under reasonable assumptions (see Methods), the change in BPND following glucose or pharmacological challenges will reflect the changes in dopamine binding to D2R-like receptors in the pancreas.

Pancreatic dopamine type 2 receptor expression overlaps anatomically virtually exclusively with insulin expression in swine pancreas (Figure S5). In support of this finding, we analyzed D2R and vesicular monoamine transporter type 2 (VMAT2) expression, by RT-PCR, in purified swine islets relative to the impure exocrine tissue by-product of the islet purification. D2R and VMAT2 transcripts were significantly enriched in islets relative to acinar tissue (Figure S6). Next, we demonstrated that dopamine mimetic ligands bind to islets in vitro (Figure S7) and show that swine islet GSIS is sensitive to dopamine suppression (Figure S8).

**Figure 5:**
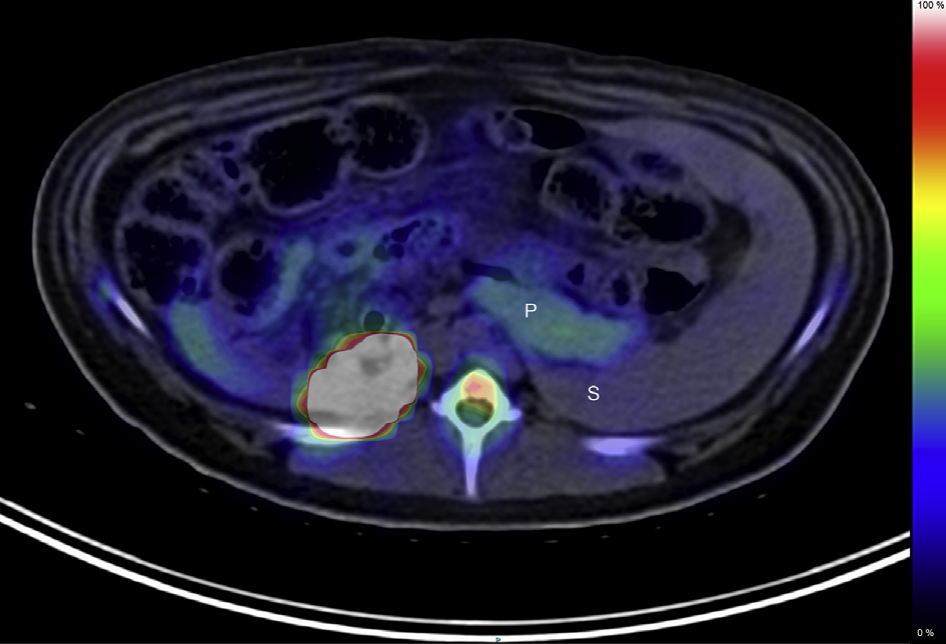
**PET scans with 18F-Fallypride visualize the porcine pancreas.** Abdominal PET/CT images (axial vial view) recorded after an intravenous bolus injection of 18F-Fallypride (153 MBq) in YMP-3. The PET portion of the image (color overlay) represent the 0–180 minute summed image. P, Pancreas, S, Spleen. Image from a representative scan of six animals.

**Figure 6:**
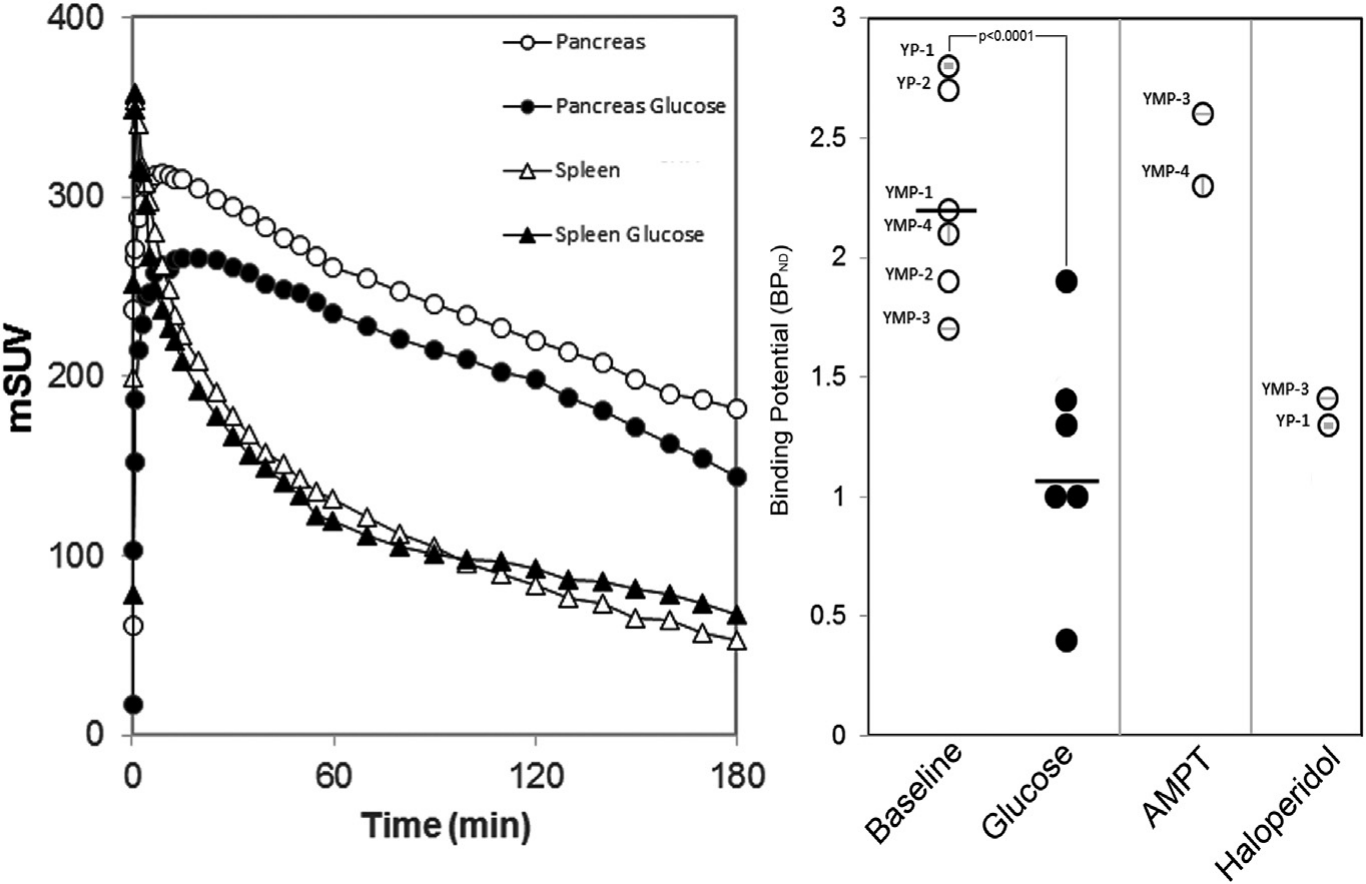
**Intravenous glucose stimulation results in loss of 18F-Fallypride tracer binding in the porcine pancreas**. Left panel, Representative pancreas and spleen time–activity curves for swine injected with a bolus of 18F-Fallypride chased with normal saline or glucose (0.5 gm/Kg). Right Panel, The binding potential with respect to the nondisplaceable compartment (BP_ND_) of 18F-Fallypride in the swine pancreas VOI was calculated using the spleen as the reference region for each experimental condition. Following i.v. glucose challenge, BP_ND_ was significantly reduced compared to fasting conditions (average fasting BP_ND_ = 2.3 ± 0.2 (S.E.M), average post i.v. glucose BP_ND_ = 1.2 ± 0.2, p < 0.0001, two-tailed paired t-test). Each separate data point is labeled with the swine ID #. BP_ND_ increased following interruption of dopamine synthesis with AMPT and decreased following D2R receptor occupancy by haloperidol (n = 2 animals for each condition).

PET quantification of total pancreatic bound18F-Fallypride provides a suitable denominator for estimating the density of D2R in the endocrine pancreas in vivo [46], [47]. PET scans with 18F-Fallypride readily visualized the porcine pancreas (Figure 5). Volumes of interest were placed over reconstructed PET images of the pancreas and the D2R free spleen reference tissue to obtain the kinetic profiles of 18F-Fallypride binding represented as time versus activity (TAC) data (Figure 4, left panel). From the TAC data (Figure 6**, left panel**), we estimated the pancreatic density of D2R (expressed as BPND) for each animal studied (Figure 6**, right panel**),. BPND refers to the ratio at equilibrium of specifically bound radioligand in the region of interest (e.g. pancreas) to that of nondisplaceable radioligand in tissue (here estimated by measurements of radioligand concentration in the receptor-free spleen reference region) and is proportional to the density of receptors available to bind radioligand in vivo [48]. In pancreas and spleen, radiolabeled metabolites likely contribute to the measured PET signal, but these should have a modest effect on the percent change in BPND following a glucose challenge. We found that glucose challenge (in the form of an intravenous glucose tolerance test, IVGTT) resulted in significant decreases (paired t-test, p < 0.0001) in the apparent D2R density, a finding consistent with in situ DA release by β-cells in response to glucose challenge. Of note, were the early changes (<10 minutes) in the post glucose challenge TAC of 18F-Fallypride, when the presence of potentially confounding metabolites is minimal [26].

We performed two additional control experiments: 1) PET scans following inhibition of dopamine synthesis by AMPT administration showed higher BP_ND_ values, consistent with the hypothesis that tissue dopamine depletion leads to greater availability of D2R to bind 18F-Fallypride; and 2) swine treated with Haloperidol, a D2R antagonist, showed a lower apparent density of D2R as reflected by the BP_ND_ (Figure 6**, far right panels**). The measured changes in the TAC profile seen after glucose stimulation (i.e. IVGTT) were unlikely due to reductions in pancreatic blood delivery of tracer as the administration of glucose is associated with a transient elevation of pancreatic blood flow [49].

## 4 Discussion

We set out to provide proof of principle that DA and L-DOPA derived from nutritional tyrosine might be involved in the defense against hypoglycemia as postulated by anti incretin hypothesis. The concept that a neurotransmitter physiologically suppresses insulin secretion as a fail-safe system defending against hypoglycemia was originally proposed by Sharp et al. [50]. For example, rodent insulin secretion is inhibited by the monoamine neurotransmitter, norepinephrine, released locally from sympathetic innervation of the pancreas or produced by the adrenal medulla and reaching islets via their unique capillary system. Neuronal modulation (via innervation of the pancreas) of β-cell insulin secretion, however, may differ in rodents and humans. Relative to the structure of mouse islets, human islets are sparsely innervated with few contacts by autonomic and cholinergic axons. Moreover, in human islets, sympathetic axons are associated with blood vessel smooth muscle cells located around the islet rather than being in direct contact with β-cells [51]. To reconcile the apparent autonomy of human islets with the known effects of autonomic stimulation on islet hormone secretion, it was suggested that “spill-over” from innervation or reduced islet microcirculation might be responsible for downstream effects of autonomic neurotransmitters on insulin secretion [52].

Previously published clinical studies provide anecdotal evidence for a direct role of GI-derived DA and L-DOPA in glucose homeostasis. These include:1) aromatic amino acid decarboxylase-deficient individuals suffer from hypoglycemia [53], 2) patients with Parkinson's disease receiving oral dopamine precursor L-DOPA display reduced insulin secretion during oral glucose tolerance testing [54]; and 3) there is an inverse correlation between circulating concentrations of C-peptide and DA [55].

Our studies demonstrate that oral TYR affects rodent insulin excursions (i.e. insulin AUCs). Plasma insulin concentration kinetics are the reflection of insulin sensitivity [56] and competing metabolic process including insulin clearance (e.g. hepatic and extrahepatic insulin clearance) and insulin production (i.e.beta cell insulin secretion). The derivative outcome measures of insulin resistance, sensitivity, and Insulinogenic index (i.e. HOMA-IR, Matsuda and Ii, respectively) calculated for the rodent experiments suggest that changes in insulin excursion are actually due to changes in beta cell insulin secretion rather than changes in insulin sensitivity. While such an interpretation of the secondary outcome measures (i.e HOMA-IR, Matsuda composite index and the Insulinogenic Index) in rodents should be viewed with caution, the overall finding, that beta cell function is affected by oral tyrosine is concordant with in vitro results and anecdotal clinical evidence. The effects of DA on insulin secretion in vitro and the observed postprandial excursions support the hypothesis that gut-derived L-DOPA and DA delivered via the circulation impact insulin secretion and postprandial glucose levels, perhaps in defense against hypoglycemia.

We previously provided evidence that DA mediates a glucose-stimulated insulin secretion (GSIS) paracrine/autocrine inhibitory circuit in human β-cells in vitro. In this circuit, DA synthesized de novo or imported by dopamine (reuptake) transporter (DAT) is stored in β-cell insulin granules by the action of the vesicular monoamine transporter type 2 (VMAT2) [12]. In our model, we hypothesized that: 1) DA and/or L-3,4-dihydroxyphenylalanine (L-DOPA) is synthesized from tyrosine, released from the gut and is present in the circulation [this current study and [7], [8]] to the beta cells; 2) L-DOPA is transformed to DA (by DOPA decarboxylase) in the beta-cells and/or; 3) DA is taken up by DAT in the beta-cells [12]; 4) DA, at basal fasting concentrations found in peripheral circulation, does not efficiently act to dampen insulin secretion, until 5) it is concentrated by the actions of VMAT2 for vesicular storage and released in high concentration near DA receptors (similar to synapse) [57]. This formulation does not exclude the direct action of circulating dopamine (following release from the GI) on beta cells in synergy with that of locally released dopamine or an indirect action of DA on somatostatin-expressing cells that have also been shown to express limited amounts dopamine receptors [58].

During GSIS, DA and insulin are released and D2R is delivered to the cell surface where it binds DA. DA, signaling through D2R, is a powerful inhibitor of GSIS. The action of DA on insulin secretion may extend to the subcellular level as well, as DA has recently been shown to complex with and stabilize the non-active insulin hexamer, shifting the equilibrium between hexamers and free insulin towards the non-active hexameric form [59]. These findings offer a mechanism for the observation that pancreatic β-cells secrete insulin in fast- and slow-release responses [60].

A weakness of our study is that the mechanisms leading to the release of intestinal L-DOPA and/or DA into the circulation remain unknown. Presumably, VMAT1-positive enteroendocrine cells and AADC-positive mucosal cells (see Figure S1) are involved in the release of DA and L DOPA, respectively. In man, oral tyrosine alone results in a transient increase of DA in plasma [34], suggesting that the release may be passive. On-the-other-hand, the finding that intestinal DA stores are depleted following MMTT relative to the fasting state suggests that release may be mechanically stimulated. Specification of these details will require experimental models not yet available to us. Another weakness of our study is that only 2 of our RYGB patients had a previous diagnosis of T2DM and that our studies were cross sectional rather than longitudinal. Further studies of these patient populations are warranted.

Bariatric surgery is an effective treatment for obesity and its associated comorbidities, including type 2 Diabetes Mellitus (T2D) [61]. Although improvement in insulin resistance secondary to weight loss and a decrease in fat mass are significant contributors to the improvement and reversal of T2D, reduced insulin resistance alone does not fully account for the efficacy of specific types of metabolic surgery. Improved β-cell function, as well as improvement in hyperglycemia, has been observed in surgeries where the upper portions of the gastrointestinal (GI) tract are bypassed and/or removed. These effects seem to be, at least in part, independent of weight loss [17]. We observed similar patterns of L-DOPA and DA excursions in rodents given a TYR/PHE free liquid meal stimulus and in RYGB patients given a complete meal stimulus. Given that RYGB surgery excludes the nutrient path from much of the tissues containing TH and AADC, we speculate the common cause to be less downstream inhibition of GSIS. Other metabolic similarities between rodents treated with Tyros 2 and mixed meal tolerance testing in RYGB patients are present. It is well documented that postprandial insulin and C-peptide excursions are increased early following RYGB [62], [63], [64], independent from weight-loss. Tyrosine depleted Tyros 2 treated rodents also showed increased insulin excursion relative to the complete mixed meal formula, a finding that is possibly relevant to the observation that hyperinsulinism is a known comorbidity of patients with Tyrosinemia type 1 treated with reduced TYR/PHE diets [65]. Recent metabolomics studies in humans clearly demonstrated that RYGB surgery is associated with lower serum concentrations of Tyrosine and/or Phenylalanine [66], [67], [68]. Further confirmation of reduced L-DOPA and DA excursions as the basis of the early post-surgical improvement in hyperglycemia observed when RYGB is used to treat T2DM associated with obesity awaits more in-depth human longitudinal studies.

We used a PET tracer study to showing glucose-dependent displacement of the dopamine analog 18F-fallypride in porcine pancreas region of interest to demonstrate a final link the proposed hypoglycemia defense circuit. However, PET quantification of total pancreatic bound 18F-Fallypride in the fasting state may have more practical applications. The amount of bound tracer might represent a surrogate measure of β-cell mass given the restriction of D2R expression to islets beta cells in the pancreas. The difference between total pancreatic bound 18F-Fallypride at baseline and total pancreatic bound 18F-Fallypride after glucose challenge might also represent a measure of β-cell function. The ratio of these two measures, a beta cell mass-normalized functional value, could have utility in the vetting of therapeutic agents for the management of T2D.

Lastly, we propose that some elements in this circuit may represent druggable targets for the manipulation of glycemia. The first step in the conversion of TYR to DA involves TH. The specific inhibitor of TH, alpha-methyl tyrosine, enhances both basal and glucose-stimulated insulin secretion in vitro [12] and in vivo [69]. We previously demonstrated that specific VMAT2 inhibitor compounds have potent in vivo glucose-lowering effects in a rat model [11], [70]. On a cautionary note, others and we have shown that certain atypical antipsychotic drugs (APDs) increase insulin secretion from islets in vitro [12], [71] while others have shown similar effects in vivo, both in rodent models [13], [72], [73] and humans [74], [75]. The common denominator among the mechanisms of action of many typical and atypical antipsychotic drugs (APD) (e.g. Risperidone, Amisulpride) is their antagonism of D2R, a distal element of our proposed gut-to-beta cell regulatory circuit. Both typical and APDs have significant metabolic side effects including significant weight gain, hypertension, and heightened risks of development of T2D [76].

## General

The authors wish to thank Drs. Bernard Herring and Piotr Witkowsky for the provision of purified porcine and human islets used in some preliminary experiments.

## Funding

These studies were supported by an award from the NIH, NIDDK (1R01DK104740) to P.E.H., the NYNORC (P30 DK026687-37) and the NIDDK DRC (P30-DK063608-15) to R.L and NIH DK072011 to J.K.

## Author contributions

These studies were conceived by R.L., A.M., and P.E.H; the isotopic enrichment studies were performed by G.W.C.; J.K led the patient recruitment and designed the clinical study; G.F. assisted with the clinical study; the development and validation of the methods used in PET analysis was performed by M.S.; G.R. purified and supplied the porcine islets used in this study; P.E.H, P.B., and A.M. performed the rodent and swine studies and analyzed the data; The original draft of the MS was prepared by P.E.H.; review & editing of the final draft was performed by all authors.
