# Supplementary material for: A role for foregut tyrosine metabolism in glucose tolerance
Source: Mol Metab. 2019 Feb 27;23:37–50. doi: 10.1016/j.molmet.2019.02.008 (PMC6479665; doi:10.1016/j.molmet.2019.02.008)
Supplement: Multimedia component 1 [file mmc1.docx]

**Supplemental Materials**

**Figure S1.** Expression of TH, AADC, VMAT1 and CGRA in the rat foregut and hindgut


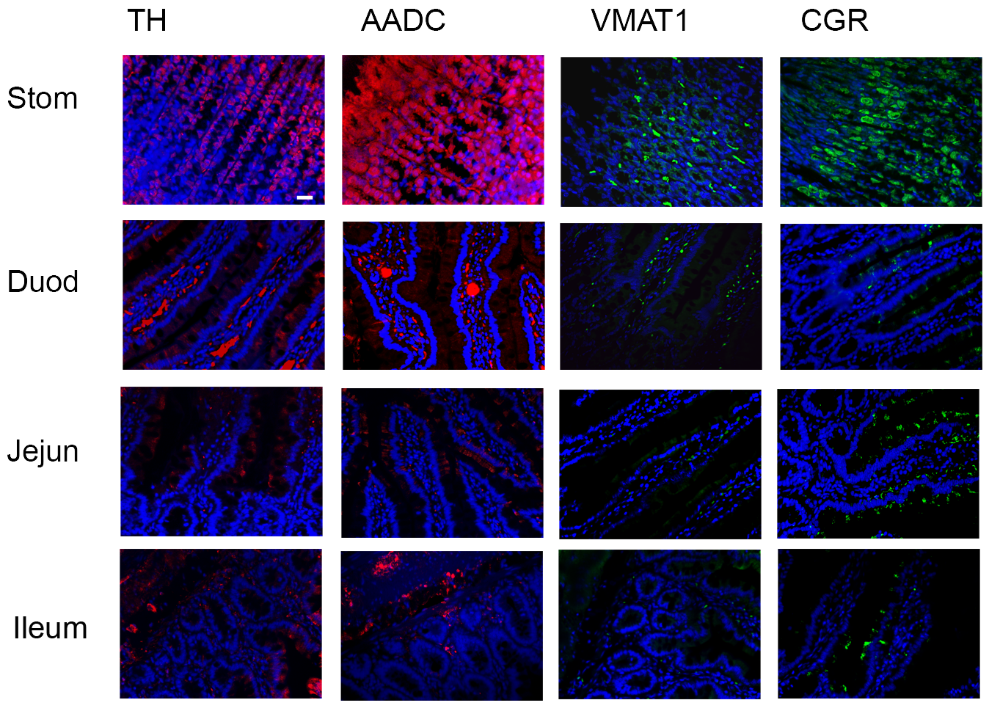


**Figure S1. Legend**. **Expression of TH, AADC, VMAT1 and CGRA in the rat foregut and hindgut**. Immunofluorescence pseudocolor micrographs of immunoreactive cells in the rat stomach (Stom) and foregut (Duod: duodenum, Jejun: Jejunum, Ileum) after labeling with antibodies to tyrosine hydroxylase (TH)(CY3-conjugated), aromatic amino acid decarboxylase (AADC) (CY3-conjugated), Vesicular monoamine transporter type 1 (VMAT1) (FITC-conjugated), and Chromogranin A (CGR) (FITC-conjugated). Nuclei counterstained with DAPI. Bar 100 μm, all images taken at the same magnification.

**Figure S2.** VMAT2 expression in rat tissues

**
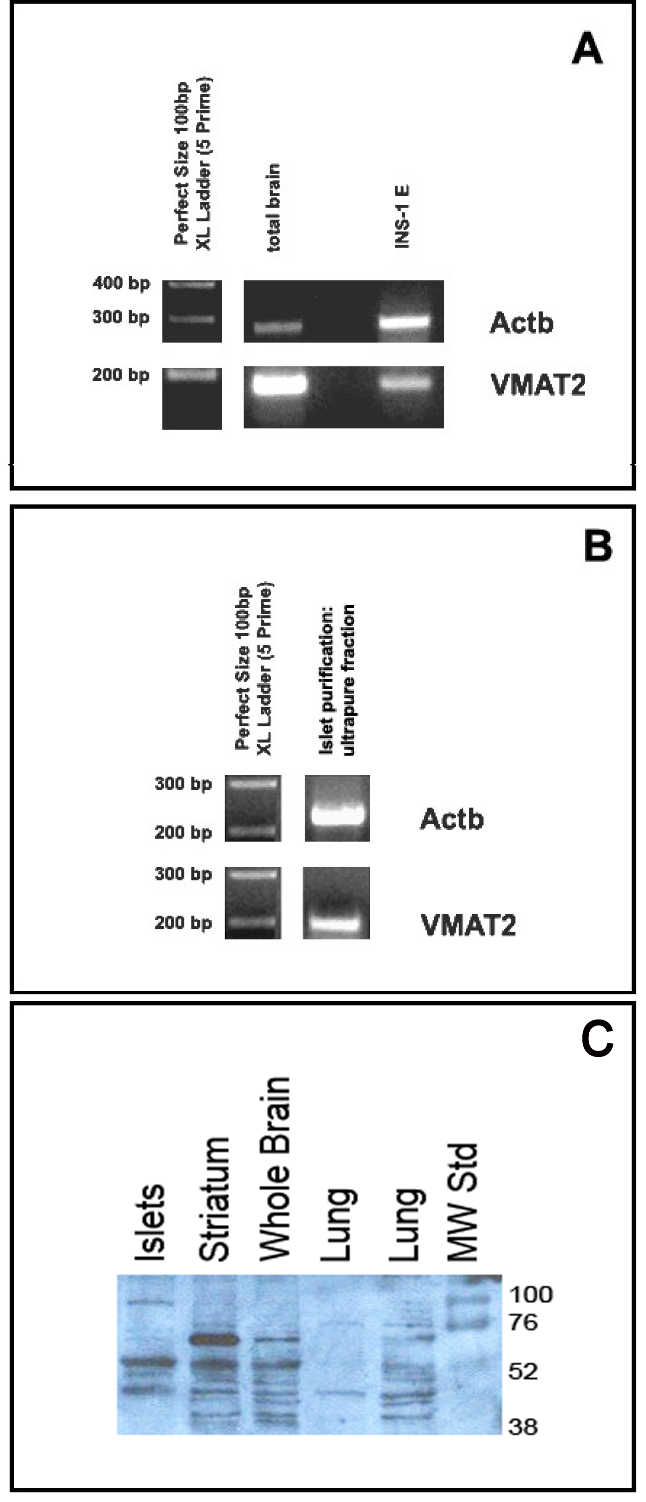
**

**Figure S2 Legend. VMAT2 expression in rat tissues.** To confirm the expression of VMAT2 in rat islets, we examined by semi-quantitative RT-PCR, the accumulation of VMAT2-specific RNA in the insulinoma cell line INS-1E compared with that in total rat brain (**Panel A**), and in rat pancreatic islets **(Panel B).** We show representative results from series of at least two experiments. In both panels the primers’ couple NewV2 was used to amplify VMAT2 transcripts (see Table S2). To amplify the Actb transcript, primers B5 and B3 were used in the experiment showed in **Panel A** and the primers B5 and AUAP were used in the experiment showed in **panel B** (see Table S2). The expression of VMAT2 proteins were probed for in the indicated Lewis rat tissue with specific antibodies (**Panel C**).Similar patterns of immunoreactivity (at 55 and 45 kDa) are shared between rat islets and brain.

**Figure S3.** Exogenous tyrosine affects oral glucose tolerance in the Goto-Kakazaki

rodent model of T2DM


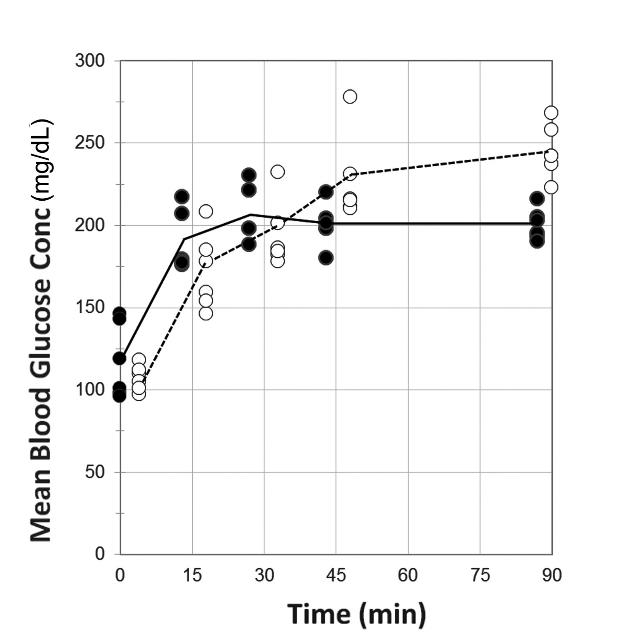


**Figure S3. Legend**. **Exogenous tyrosine affects oral glucose tolerance in the Goto-Kakazaki**

**rodent model of T2DM**. Male (n=6, weight range 322-354 gm, 11-12 weeks old) Goto-Kakazaki were gavaged with a 50% glucose solution with and without added L- tyrosine (12 mg/ml). Measurements of whole blood glucose concentrations were made in serial blood samples obtained from the tail. Solid (OGTT) and dashed lines (OGTT W/TYR) connect the mean glucose concentrations at each time point tested. The excursions were significantly different (p<0.05) by a repeated measure ANOVA.

**Figure S4.** Human islets and INS1E cells produce DA or L-DOPA from metabolic precursors.


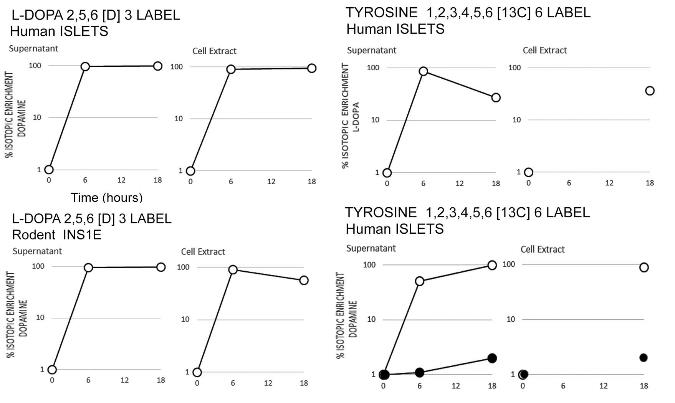


**Figure S4. Legend**. **Human islets and INS1E cells produce DA or L-DOPA from metabolic precursors**. Islet and INS-1E cell cultures were treated with stable isotope labeled TYR and or LDOPA for the indicated amount of time in the presence (open circles) or absence (black circles) of 10 μM pargyline, 10 μM Moclobemide and 10 μM GBR 12909. Cell extracts and cultures supernatants were analyzed for the presence of stable isotope labeled L-DOPA and DA by LC-ESI-MS/MS. Results are averaged from duplicate measurements performed in two separate experiments. All enrichment values at times greater than 0 were significantly different (p<0.05) from the time zero value by a Mann-Whitney U test.

**Figure S5.** Insulin and D2R expression overlap in the porcine pancreas


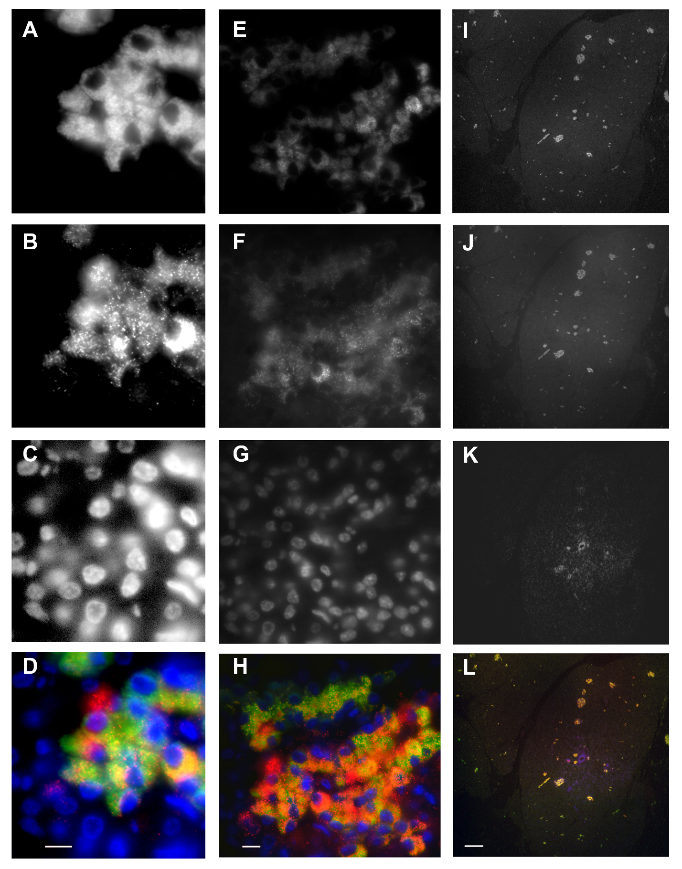


**Figure S5. Legend**. **Insulin and D2R expression overlap in the porcine pancreas**. Double immunofluorescence staining for insulin and D2R in porcine pancreas sections. Panels A, C, E and G, (high magnification, bar 10 μm), Panels B, D, F, and H, (low magnification, bar 200 μm). Panels A-B, Anti insulin, FITC label. Panel C-D, Anti-D2R, CY3 label. Panel E-F, DAPi nuclear stain. Panel G-H, Composite pseudocolor image from merged FITC, CY3 and DAPI channels.

**Figure S6.** Expression of D2R in pig islets and semipurified acinar tissue


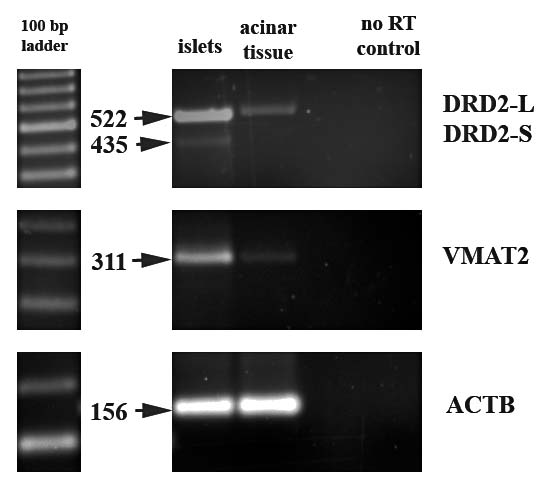


**Figure S6. Legend**. **Expression of D2R in pig islets and semipurified acinar tissue**. To confirm the expression of D2R in pig islets and semipurified acinar tissue, we examined the expression of D2R at the transcript level in both the enriched islets fraction and the exocrine tissue enriched fraction from each preparation. Using semi-quantitative PCR and primers specific for both the Long (DRD2-L) and the Short (DRD2-S) isoforms of D2 dopamine receptor (**Table S1**) we amplified cDNA prepared from the RNA of purified islets and of the corresponding exocrine tissue. As a control, we also analyzed the expression of VMAT2, a protein used as a specific biomarker of pancreatic beta cells . Representative results from series of two experiments

**Figure S7.** Porcine islets cells bind and internalize the dopamine receptor probe DnsykD-1





**Figure S7. Legend**. **Porcine islets cells bind and internalize the dopamine receptor probe DnsykD-1.** Fluorescence imaging (385nm excitation/515 nm emission) of live porcine islets cells treated with DnsykD-1. Serial images of dispersed islet cells taken at Panel A, 15 minutes, Panel B, 30 minutes and Panel C 45 minutes after addition of 10 μM DnsykD-1 to cultures. Panels E through D. Cultures imaged at 15, 30 and 45 minutes after addition of 10 μM DnsykD-1 and 1 mM Haloperidol. Bar 4 μm.

**Figure S8.** GSIS in porcine islets is sensitive to exogenous DA

**

**

**Figure S8. Legend.** **GSIS in porcine islets is sensitive to exogenous DA**. Static incubation experiments were performed using approximately 50 I.E.Q. per well of islets distributed in culture wells in duplicate. Each pool of Yorkshire islets was tested against 3.0mM glucose, 17 mM glucose, and 17mM glucose with indicated concentration of dopamine. After a 1-hour incubation, the insulin concentration (technical replicates of three) in the incubation buffer in each well was determined by ELISA and normalized to the exact number of plated islets. The mean normalized insulin concentration for duplicate wells is indicated. Error bars indicate the SEM. * indicates a significantly difference from 3 mM basal condition. ** indicates a significant difference from 17 mM glucose stimulated conditions (p < .05). From a representative experiment in a series of two.

| Table S1 - Reagents and Equipment | | |
| --- | --- | --- |
| RESOURCE | SOURCE | IDENTIFIER |
| Antibodies |  |  |
| Anti-Tyrosine Hydroxylase Antibody (Rabbit) | Boster | PB 9449 or RRID : AB_2728729 |
| Anti-DOPA Decarboxylase Antibody (Rabbit) | Boster | A01374-1 or RRID : AB_2728730 |
| SLC18A1 antibody - middle region (Rabbit) | Aviva | RRID:AB_2190655 |
| SLC18A2 antibody - C-terminal peptide sequence from rat | EMDMillipore | AB1598P or RRID:AB_2285927 |
| Anti-Chromogranin A, Clone LK2H10 (Mouse mAb) | EMDMillipore | RRID:AB_11213294 |
| Anti-Dopamine D2 Receptor antibody(Rabbit) | Abcam | ab150532 or RRID:AB_2753119 |
| Anti-Insulin/Proinsulin antibody(Mouse mAb) | Abcam | ab105435 or RRID:AB_10861426 |
| Goat anti Rabbit IgGs-Alexa fluor 430 | ThermoFisher | RRID:AB_2534111 |
| Goat Anti-Mouse IgG H&L - FITC | Abcam | 6785-1 |
| Goat anti-Rabbit IgGs -Cy3 | Boster | BA1032 |
| Reagents for RNA preparation and analysis. |  |  |
| RNeasy Lipid Tissue Mini Kit | Qiagen, Valencia, CA | 74804 |
| VILO cDNA synthesis kit | Life Technologies | 11754050 |
| 3' RACE System for Rapid Amplification of cDNA Ends | ThermoFisher | 18373019 |
| AccuPrime™ Pfx SuperMix | ThermoFisher | 12344040 |
| GelRed™ Nucleic Acid Gel Stain | Biotium | 41003 |
| PerfectSize 100bp XL Ladder | 5Prime | 2500340 |
| Biological Samples |  |  |
| Purified human cadaveric pancreatic islets | IIDP | RRID:SAMN08768973 and RRID:SAMN08768991 |
| Purified porcine islets | Alberta Diabetes Institute, Univ. of Alberta |  |
| Purified rodent islets | In house |  |
| Mixed Meal Stimuli |  |  |
| Optifast 800 | Novartis | https://www.optifast.com/Pages/products/optifast-800-ready-to-drink-shakes.aspx |
| Ensure Original | Abbott | https://ensure.com/nutrition-products/ensure-original |
| Tyros 2 | Mead Johnson | https://www.meadjohnson.com/pediatrics/us-en/product-information/products/metabolics/tyros-2 |
| Chemicals |  |  |
| α-Methyl para Tyrosine | Sigma-Aldrich | M8131 |
| Alumina Oxide, activated, acidic, Brockmann I | Sigma-Aldrich | 199966 |
| All L-amino acids | Sigma-Aldrich | various |
| [L-tyrosine (Ring-13C6, 99%)](http://shop.isotope.com/productdetails.aspx?itemno=CLM-1542-PK) | Cambridge Isotope Labs | CLM-1542-PK |
| L-tyrosine (13C9,99%,15N 99%) | Cambridge Isotope Labs | CNLM-439-H-0.5 |
| Dopamine:HCL (2-(3,4-Dihydroxyphenyl)-Ethylamine:HCl) (Ring-13C6, 99%) | CDN Isotopes Pointe-Claire, Quebec  Canada H9R 1H1 | C-334 |
| L-DOPA (Ring-D3, 98%) | CDN Isotopes | D-1570 |
| Glucose | Sigma-Aldrich | G7528 |
| DnsylD-1 | Sigma-Aldrich | 95199 |
| Sodium 1-Octanesulfonate | Nacali Tesque | https://www.eicomusa.com/store/hplc-ecd/sos/ |
| Citric Acid, Anhydrous | Fisher Scientific | AC385850010 |
| Acetic acid, sodium salt, anhydrous | Fisher Scientific | AC424255000 |
| Ethylenediaminetetraacetic acid, tripotassium salt dihydrate | Fisher Scientific | AC301601000 |
| Norepinephrine bitartrate | Sigma-Aldrich | 1468501 |
| L-DOPA | Sigma-Aldrich | PHR1271 |
| 3,4-Dihydroxyphenylacetic acid (DOPAC) | Sigma-Aldrich | 11569 |
| Dopamine hydrochloride | Sigma-Aldrich | PHR1090 |
| 5-Hydroxyindole-3-acetic acid (5-HIAA) | Sigma-Aldrich | H8876 |
| Isoproterenol hydrochloride | Sigma-Aldrich | 1351005 |
| 4-Hydroxy-3-methoxyphenylacetic acid (HVA) | Sigma-Aldrich | 69673 |
| 3-Methoxytyramine hydrochloride (3-MT) | Sigma-Aldrich | 65390 |
| 5-Hydroxytryptamine hydrochloride (5-HT) | Sigma-Aldrich | H9523 |
| HEPES | Sigma-Aldrich | H4034 |
| Sodium Pyruvate | Sigma-Aldrich | P5280 |
| L-Glutamine | Sigma-Aldrich | G5792 |
| 2-Mercaptoethanol | Sigma-Aldrich | M6250 |
| Penicillin G | Sigma-Aldrich | P3032 |
| Streptomycin sulfate | Sigma-Aldrich | S9137 |
| Pargyline hydrochloride | Sigma-Aldrich | P8013 |
| GBR12909 dihydrochloride | Sigma-Aldrich | D052 |
| Moclobemide | Sigma-Aldrich | M3071 |
| Haloperidol | Sigma-Aldrich | H1512 |
| DMSO | Sigma-Aldrich | D2650 |
| Perchloric Acid | Fisher Scientific | AC452850010 |
| Bovine Serum Albumin (BSA) Fatty Acid-free | Fisher Scientific | BP9704100 |
| **Tissue Culture** |  |  |
| RPMI 1640 Medium, No Phenol Red | Fisher Scientific | 11-835-030 |
| RPMI 1640 Medium | Fisher Scientific | 11-875-085 |
| RPMI 1640 Med. w/o Amino Acids | Fisher Scientific | NC0750940 |
| Fetal Bovine Serum | Atlanta Biologicals | S11150H |
| 0.25% Trypsin, 0.1% EDTA in HBSS w/o Calcium, Magnesium and Sodium Bicarbonate | Fisher Scientific | MT25053CI |
| **Radiochemicals** |  |  |
| 18F-Fallypride | PETNET Solutions | https://usa.healthcare.siemens.com/molecular-imaging/petnet-solutions |
| **Commercial Assays** |  |  |
| Human L-Dihydroxyphenyalanine, L-DOPA ELISA Kit | MyBioSource | MBS9301852 |
| Rat insulin ELISA Kit | ALPCO | 80-INSRT-E01 |
| Human insulin ELISA Kit | ALPCO | 80-INSHU-E01 |
| Multispecies GLP-1 ELISA | Millipore | EZGLP1T-36K |
| Western blotting ECL kit | Pierce | 35066 |
| **Experimental Models: Cell Lines** |  |  |
| Ins-1E | AddexBio | C0018009 or RRID:CVCL_0351 |
| **Experimental Models: Organisms/Strains** |  |  |
| Lewis rats | Charles River | 004 |
| Goto Kakazaki rat | Taconic | GK-M |
| Yorkshire pigs | Animal Biotech Industries | http://www.animalbiotech.com/research-swine/live-animal-models/ |
| Yucatan mini Pigs | Sinclair BioResources | http://www.sinclairbioresources.com/miniature-swine/yucatan/ |
| **Software and Algorithms** |  |  |
| **MEDCALC 17.7.2** | MedCalc Software | https://www.medcalc.org/ |
| **PMOD 3.0** | PMOD TECHNOLOGIES LLC | https://www.pmod.com/web/ |
| **SYNGO FASTVIEW** | Siemens Medical Solutions | https://static.healthcare.siemens.com/siemens_hwem-hwem_ssxa_websites-context-root/wcm/idc/groups/public/@us/@healthit/documents/download/mda2/mtg3/~edisp/vx57n-03253353.zip |
| **Alphaview SA software v.3.4.0.0** | Protein simple, San Jose, California | https://www.proteinsimple.com/imaging-crunch-some-numbers |
| **Infinity Analyze 6.5.4** | Lumenera | https://www.lumenera.com/support/microscopy/drivers-downloads.html |
| **Other** |  |  |
| Biograph mCT PET/CT camera | Siemens Medical  Solutions USA, Inc. | Malvern, PA |
| GeneQ thermal cycler | Bulldog Bio | http://www.bulldog-bio.com/thermalcyclers.html |
| FluorChem M | Protein simple | https://www.proteinsimple.com/fluorchem_m.html |
| Alphatrak glucometer | Zoetis | https://www.zoetisus.com/products/dogs/alphatrakmeter/alphatrak-veterinarians.aspx |
| LC-MS/MS | Shimazu UFLCXR, QTRAP 6500 | 7102 Riverwood Drive,  Columbia, MD 21046 |
| HPLC-EDC HITEC 500 and software suite | EICOM | https://www.eicomusa.com/hplc-ecd/htec-500/ |
| Synergy 2 | Biotek | https://www.biotek.com/products/detection-multi-mode-microplate-readers/synergy-2-multi-mode-reader/ |
| Mini-Beadbeater-16 | Biospec products | 607 |
| Beadbug prefilled tubes (2 ml) | Sigma-Aldrich | 763799 |
| Nylon MicroSpin Centrifuge Filters with PP Housing - 0.20µm | Analytical Sales and Service, Inc | 8505-00 |
|  |  |  |

**Table S2 – List of the primers used in this paper**

| Species Specificity | Transcript Name | Primer Name | Primer Sequence 5’ > 3’ | Expected Fragment size |
| --- | --- | --- | --- | --- |
| Pig | ACTB | PigACTB_F | ccctggagaagagctacgag | 156 |
|  |  | PigACTB_R | CGTCGCACTTCATGATGGAG |  |
|  | VMAT2 | P2_F | CTGCTCATCGCCAGGTCGCT | 311 |
|  |  | P2_R | AGCGTGGTTAGCGGTGTCCC |  |
|  | D2R | PigD2_2_F | CCATCGTCTGGGTCCTGTCT | 522 Long D2R  435 Short D2R |
|  |  | PigD2_2_R | CGTGTCCGTTCTTCTCTGGT |  |
| Rat | Actb | B3 | CCTAGAAGCATTTGCGGTGCACGATG | 285 |
|  |  | B5 | TCATGAAGTGTGACGTTGACATCCGT |  |
|  |  |  |  | ~ 390 |
| No specificity |  | AUAP | included in the 3´ RACE System |  |
| Rat | VMAT2 | NewV2_F | AGCGTGTACACAGATGATGAGG | 193 |
|  |  | NewV2_R | ACGGCTGGAGCACAAAGA |  |
